# Higher self-assessed subjective social status is associated with worse perception of others’ emotions

**DOI:** 10.1038/s41598-025-01493-2

**Published:** 2025-05-17

**Authors:** Victoria K. Lee, Mel W. Khaw, Rachel E. Kranton, Scott A. Huettel

**Affiliations:** 1 Department of Psychology and Neuroscience, Duke University, Durham, NC 27708 USA; 2 Department of Economics, Duke University, Durham, NC 27708 USA; 3 Center for Cognitive Neuroscience, Duke University, Durham, NC 27708 USA

**Keywords:** Emotion perception, Empathic accuracy, Social cognition, Subjective social status, Social class, Human behaviour, Social behaviour, Emotion

## Abstract

The ability to accurately perceive others’ emotions is arguably critical for successful social interaction and may facilitate upward social mobility through personal and career advancement. Yet, prior research suggests that individuals of lower social status are better at perceiving others’ emotions. These competing viewpoints lead to the question of whether emotion perception ability—often referred to as empathic accuracy—shapes or is shaped by social status. In a preregistered experiment (n = 1197), we tested these alternate perspectives and found a robust negative relationship between individuals’ self-reported social status and behavioral measures of emotion perception. These effects were limited to emotions expressed by an individual actor and did not extend either to emotions expressed in a group or to similar judgments in a nonsocial control context. In addition, we found preliminary evidence that self-assessed increases in social status over the lifespan were also associated with worse emotion perception. These patterns support the perspective that social status shapes emotion perception abilities, but importantly, this relationship depends on one’s subjective sense of their status, both in comparison to others and in evaluations of one’s own lifespan trajectory.

## Introduction

Humans are highly social beings who thrive on interactions. Core to these interactions is the ability to understand the perspectives of others, including their mental and affective states^1,2^. However, variation in social cognition, including the ability to identify and perceive the emotions of others (i.e., *empathic accuracy*), has been linked to systematic differences among individuals in society. More specifically, lower social status has been associated with engagement of the mentalizing neural network^3^, social attention^4^, compassion^5^, mutual understanding^6^, perspective taking^7^, and, of particular interest here, emotion perception^7–10^. For those who possess relatively few resources, these social cognitive abilities support survival in a relatively uncertain and threat-filled context^10^ that encourages social vigilance^11^, cooperative solutions to daily tasks^12^, and greater prosocial behavior^.^^13–15^.

Yet, social cognition has demonstrable benefits for personal and economic success in an interconnected society^16^. Socially competent individuals with higher emotional intelligence may be more successful in their careers, allowing for upward social mobility^17,18^. Some research identifies a positive relationship between emotion perception and social status: individuals with higher socioeconomic status (SES) score higher on emotion perception tasks, while lower SES individuals perform worse in emotion identification and discrimination tasks in larger, more diverse samples^19–21^. Furthermore, children with higher trait emotional intelligence are more likely to be socially accepted and perform well in school^22^, suggesting that social cognitive abilities can catapult individuals along pathways that lead to increased social status.

How can these opposing perspectives – one suggesting that lower social status generates conditions that improve emotion perception and another suggesting that emotion perception abilities potentiate advancement to higher social status – be reconciled? In a preregistered study, we addressed this question in two ways. First, we collected subjective and objective measures of social status and examined their individual and combined predictive power on emotion perception abilities while controlling for known confounds of emotion perception (e.g., age, gender, race/ethnicity, political affiliation, agreeableness, and sense of power). This design allowed us to rule out possible differences in how social status is measured (i.e., subjectively versus objectively), which is particularly important because subjective and objective measures may assess different aspects of social status (e.g., a college student who comes from a wealthy family may nevertheless report a very low income). Second, we asked participants to self-report their current and childhood social status to distinguish between effects associated with *change in social status* and effects associated with *current social status*. Specifically, if emotion perception leads to social status (e.g., if having greater social skills allows you to move up in social status), we would expect a positive relationship such that increases in social status are associated with better emotion perception ability and decreases are associated with worse emotion perception ability. However, if social status leads to emotion perception ability (e.g., if lower social status leads to greater interdependence & social attunement), we would expect a negative relationship such that increases in status are associated with worse emotion perception ability and decreases in social status are associated with better emotion perception ability.

**Fig. 1:**
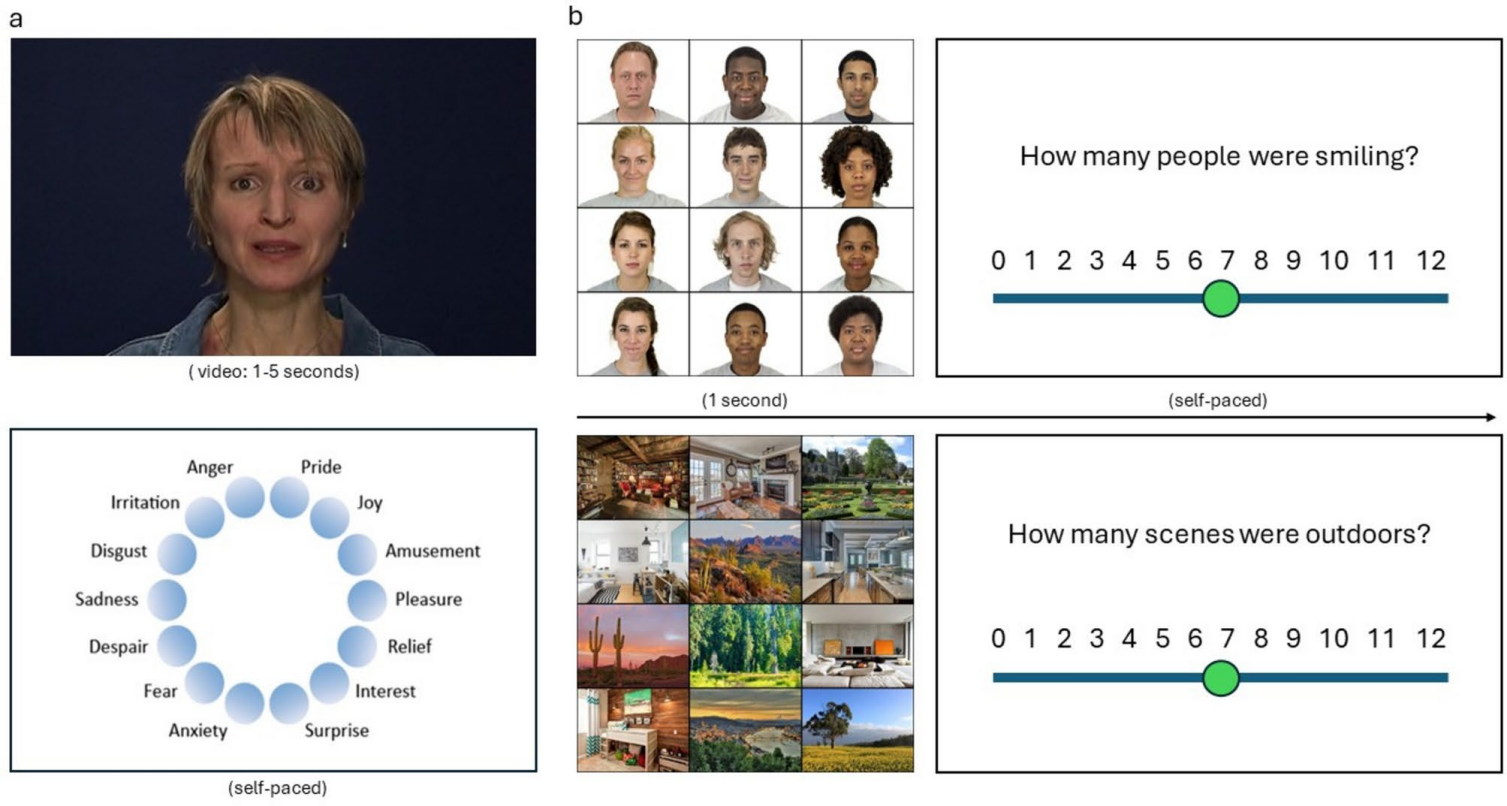
Emotion perception tasks. Participants completed two emotion perception tasks, presented in a randomized order. (**a**) Individual emotion perception task (Geneva Emotion Recognition Test—Short form, GERT-S). Participants viewed a 1–5 s video clip of an actor speaking meaningless syllables. Then participants indicated the emotion expressed by the actor by selecting 1 out of 14 possible emotions. (**b**) Group emotion perception task (Ensemble Emotion Task, EET). Participants viewed an ensemble of images for one second (either faces or scenes; trials intermixed) and then indicated the number of smiling faces or outdoor scenes. Note that here we simplify the decision screen for the EET for readability; see study repository for exact display materials.

We investigated these effects in two common situations: judgment of a single individual’s emotions and determining emotions among a group of individuals. Research shows that people are able to identify emotions quickly and accurately for individuals^23^ and for groups^24^, but it is not known whether status has similar effects in these two settings. For individual perception, participants judged the emotion expressed by an actor in a short video (GERT-S^25^); this task overcomes limitations of previous paradigms (e.g., the Reading the Mind in the Eyes task, RMET) that introduce unintended biases associated with social status^20,26^. For example, whereas the RMET uses static, eyes-only images and complex vocabulary response options that allow individuals with higher IQ, education, and specific cultural background to perform well^20,26^, the GERT-S offers dynamic visual and audio cues from an actor and asks participants to select from 14 common emotions that vary in valance and arousal^25^. In another task, participants indicated the proportion of smiling faces presented in an ensemble^27^. We describe this as group emotion perception, though we emphasize that the individuals in the ensemble task are not interacting with each other and that emotion perception is limited to just one emotion (happiness/smiling). We additionally included a parallel non-social task (i.e., judgment of indoor and outdoor scenes) to control for overall perceptual and cognitive abilities (See Fig. 1).

Our experimental design extends prior work examining the relationship between social status and emotion perception. First, we utilized an inclusive battery of relevant objective and subjective social status measures used in prior studies; our analyses tested their individual and combined predictive power of emotion perception performance. Second, we go beyond examination of current social status to include self-assessed changes in status from childhood to adulthood; this allows us to determine whether change in status provides information about emotion perception abilities independent from that of current status and to gather evidence in support of one of the perspectives described above. Third, we paid participants for accuracy in their performance to ensure incentive compatibility^28^. Fourth, we used a large sample that was stratified by prescreening questions to include participants with a broad range of subjective social status. Last, we established the specificity of our findings by examining alternative ways of characterizing both social status and emotion perception ability.

## Results

Data were collected and analyzed as outlined in our preregistration (see Methods). Any analyses that extended beyond the preregistration are explicitly described as “exploratory” hereafter. These follow-up exploratory analyses examined moderators, boundary conditions, and robustness checks of observed significant effects and explored the extent to which subjective social status drives these effects. In addition, exploratory analyses examined whether the change in social status is related to emotion perception in a way that is different from current social status.

### Performance in the emotion-perception tasks

Participants completed two emotion perception tasks. In the individual emotion-perception task (GERT-S), participants viewed short video clips and selected the emotion expressed by the actor in the video from a list of 14 emotions. Participants selected the correct emotion on 59.7% of trials (95% CI = [59.1, 60.4]); if simply guessing, participants would have had a 1 in 14, or 7%, chance of being accurate on each trial. In the group emotion-perception task (EET), participants briefly viewed (1 s) displays of an ensemble of 12 faces and estimated the proportion of smiling individuals. Participants were accurate 41.2% (95% CI = [40.5, 41.9]) of the time with a typical precision of 1.37 units (95% CI = [1.34, 1.4]). In the nonsocial control perception task with indoor and outdoor scenes (accuracy: 48.4%, 95% CI = [47.6, 49.2]; Precision: M = 1.2, 95% CI = [1.16, 1.23]) performance was better than performance in the EET for both accuracy [*t*(1196) = −17.4, *p* < 0.0001] and precision [*t*(1196) = 9.78, *p* < 0.0001]. See Supplementary Section S1 for additional differences in performance by demographic and Supplementary Table S1 for correlations between performance in each of the emotion perception tasks.

### Subjective social status depends on objective markers of social status

Participants reported objective and subjective measures of social status. Objective measures included participants’ income and education level, as well as household income. For regressions that include objective social status measures, we separated household income into income for self and income for others in the household (i.e., calculated as household income minus income for self; see Methods). Subjective measures included perceived ranking on a socioeconomic ladder (MacArthur Subjective Social Status, abbreviated MSSS^29,30^) and ratings of statements related to SES (Subjective SES Scale, abbreviated SSS^31^). While we adopt the more general term “social status” throughout the manuscript to reflect the linear ranking of one’s position within society (e.g., income, education, placement on socioeconomic ladder), we also measured more categorical classifications associated with “social class” (e.g., middle class), as these terms are often used interchangeably within psychology^8^. This included the Subjective Social Class measure (SSC^32^), in which participants are asked which of five social classes they belong to: poor, working class, middle class, upper middle class, and upper class. See Supplementary Table S1 for correlations between these status measures.

For all three subjective social status measures (i.e., MSSS, SSC, and SSS), we observed significant positive relationships with objective social status measures (preregistered H1). As income for self, income for others in the household, and education increased, subjective measures of social status also increased (MSSS: income *b* = 0.47, *t* = 23.54, *p* < 0.0001, 95% CI [0.43, 0.5]; income for others in household *b* = 0.36, *t* = 19.7, *p* < 0.0001, 95% CI [0.33, 0.4]; education *b* = 0.19, *t* = 6.77, *p* < 0.0001, 95% CI [0.13, 0.24]; see Supplementary Table S2 for SSC and SSS). However, these correlations were not perfect – the objective measures of social status only explained approximately 38–55% of the variance in subjective social status measures (see Supplementary Table S3), suggesting that an individual’s subjective social status is determined by more than just income and education.

To further understand the relationship between subjective and objective measures of social status, we examined the breakdown of household income by MSSS. As demonstrated in Table 1, there was a positive relationship between subjective and objective social status indicators; the majority of people who indicated they are lower on the MSSS ladder had a lower household income, while the majority of people who indicated they are higher on the MSSS ladder had a higher household income. However, there was also variation in these responses, again suggesting that there may be multiple factors that influence one’s subjective social status.

**Table 1:** Household income by MacArthur Subjective Social Status.

	MacArthur Subjective Social Status						
	2	3	4	5	6	7	8
Decile 1 (less than $15,000)	34.2	15.3	8.4	5.4	2	2.1	0.9
Decile 2 ($15,000—$25,000)	25.2	19	10.5	4.8	3.9	2.6	0
Decile 3 ($25,001—$40,000)	23.6	31	25.1	9.6	6.9	4.7	3.8
Decile 4 ($40,001—$50,000)	8.1	14.4	12	10.2	8.8	3.1	2.8
Decile 5 ($50,001—$70,000)	5.7	12	19.4	23.5	16.7	11	4.7
Decile 6 ($70,001—$85,000)	0.8	5.1	10.5	10.8	15.2	8.4	4.7
Decile 7 ($85,001—$110,000)	0	1.4	8.4	22.3	21.6	17.8	15.1
Decile 8 ($110,001—$140,000)	1.6	0.5	3.7	7.8	11.3	19.9	14.2
Decile 9 ($140,001—$200,000)	0	0.9	1.6	4.2	10.8	18.3	24.5
Decile 10 (greater than $200,000)	0.8	0.5	0.5	1.2	2.9	12	29.3
N	123	216	191	166	204	191	106
Mean (rounded to nearest 1,000)	29,000	38,000	53,000	73,000	88,000	114,000	142,000

Note : Household income is reported in deciles drawn from the US income distribution in 2021. Ranges reported in parentheses correspond to response options presented to participants. MacArthur Subjective Social Status is reported as the ladder rung selected by participants (ranging from 1–10; rungs 1, 9, and 10 were excluded from analyses; see Methods). Each cell contains the percent of participants reporting, with columns summing to 100%. Average household income was calculated after converting each participant’s selected range to a single number (e.g., Decile 1 = $15,000, Decile 2 through 9 = the mean value for that range, Decile 10 = $200,000).

### Lower subjective social status is associated with better perception of other individuals’ emotions

Replicating previous literature, we observed negative correlations between social status and emotion perception for both subjective (MSSS; *r*(1195) = −0.06, *p* = 0.03, 95% CI = [−0.12, −0.01; preregistered H2a) and objective (income for self;* r*(1195) = −0.09, *p* = 0.001, 95% CI = [−0.15, −0.04; preregistered H2b) measures (see Supplementary Table S1). We also observed a positive correlation between emotion perception and income for others in the household (*r*(1195) = 0.08, *p* = 0.01, 95% CI = [0.02, 0.13]); however, this effect was linked to a difference between individuals with other incomes in the household compared to those with no other household income (see Supplementary Section S2 for details).

We next evaluated whether these effects were robust to controls for other factors known to influence emotion perception abilities (e.g., age, gender, race/ethnicity, political affiliation, agreeableness, sense of power; preregistered H3; Table 2). We again observed a significant effect of subjective social status (MSSS) on emotion perception (GERT-S), such that higher social status participants performed worse on GERT-S (*b* = −0.45, *t* = −2.53, *p* = 0.01, 95% CI = [−0.8, −0.1]; preregistered H3, restricted model). Additionally, controlling for objective measures of social status (e.g., income for self, income for others in the household, education) in our full model did not eliminate the effect of subjective social status on emotion perception (*b* = −0.62, *t* = −2.5, *p* = 0.01, 95% CI = [−1.1, −0.13]; preregistered H3), indicating that subjective social status provided unique explanatory power.

**Table 2:** Subjective social status negatively predicts emotion perception (preregistered H3) for individuals, but not groups.

	Individual emotion perception task (GERT-S)		Group emotion perception task (EET)	
	Restricted model	Full model	Restricted model	Full model
Intercept	69.9***	68.18***	1.05***	1.07***
	[63, 76.81]	[61.21, 75.15]	[0.74, 1.35]	[0.76, 1.37]
Subjective social status (MSSS)	−0.45*	−0.62*	−0.004	0.01
	[−0.8, −0.1]	[−1.1, −0.13]	[−0.02, 0.01]	[−0.01, 0.03]
Age	−0.06*	−0.05*	0.01***	0.01***
	[−0.11, −0.01]	[−0.1, −0.001]	[0.01, 0.01]	[0.005, 0.01]
Female	1.77**	1.3^t^	−0.04	−0.03
	[0.46, 3.08]	[−0.05, 2.64]	[−0.1, 0.02]	[−0.09, 0.03]
White	2.19**	2.12**	−0.02	−0.02
	[0.68, 3.7]	[0.61, 3.62]	[−0.09, 0.04]	[−0.09, 0.05]
Democrat	1.87**	1.77**	−0.04	−0.04
	[0.56, 3.18]	[0.45, 3.08]	[−0.1, 0.02]	[−0.1, 0.02]
Agreeableness	−0.29	−0.38	0.02	0.02
	[−1.08, 0.51]	[−1.17, 0.42]	[−0.02, 0.05]	[−0.02, 0.05]
Sense of Power	−2.09**	−2.14**	0.01	0.01
	[−3.56, −0.62]	[−3.6, −0.68]	[−0.05, 0.08]	[−0.05, 0.08]
Income for self		−0.24		−0.01
		[−0.65, 0.17]		[−0.03, 0.01]
Income for others in household		0.39*		−0.02*
		[0.03, 0.75]		[−0.03, −0.001]
Education for self		0.59*		−0.002
		[0.11, 1.07]		[−0.02, 0.02]

Note : ^*t*^*p* < 0.10, * p < 0.05, ** p < 0.01, *** p < 0.001*; *Table contains estimate, significance, [95% CI].

While we observed the above effects for the MSSS scale, we did not observe significant relationships between GERT-S and either of the other subjective social status measures (SSC or SSS; see Supplementary Table S4). However, both MSSS effects (H3 restricted and full models) survive a Bonferroni multiple comparison correction, using an alpha = 0.0167 to correct for the three tests of subjective social status.

### Subjective features of social status underlie the negative relationship between social status and emotion perception for individuals

In our preregistered analyses outlined above, we saw significant effects for both subjective and objective social status. To assess the independent variance explained by subjective social status, we performed an exploratory analysis using hierarchical regression. Our initial set of control variables (demographics, agreeableness, and sense of power) explained 2.9% of the variation in emotion perception (GERT-S), *F*(6,1189) = 5.88, *p* < 0.0001. Adding objective measures of social status (income for self, income for others in the household, education for self) explained an additional 1.2% of the variation (Δ*R*^*2*^ = 0.012), *F*_change_ (3, 1186) = 4.78, *p* = 0.003). Finally, adding subjective social status (MSSS) contributed an additional 0.5% of variation explained (Δ*R*^*2*^ = 0.005, *F*_change_ (1, 1185) = 6.27, *p* = 0.01), suggesting that there was a small but unique explanatory contribution of subjective social status above that provided by control variables and measures of objective social status.

To further explore the unique effects of subjective social status on emotion perception, we examined an alternative scoring of the MSSS score that characterized the extent to which participants used similar scales in their subjective assessments on the MSSS relative to those who choose the same subjective social class in the SSC measure (e.g., two middle class individuals rate themselves as 4 vs 7 on the MSSS; see Methods). As shown in Supplementary Table S5, when controlling for known confounds of emotion perception and objective measures of social status, individuals who rated their social status relatively higher than similar others tended to have worse emotion perception abilities than those who rated their subjective social status relatively lower (*b* = −0.85, *t* = −2.69, *p* = 0.01, 95% CI = [−1.47, −0.23]).

### Preliminary evidence for a negative relationship between change in social status over the lifespan and emotion perception for individuals

We examined the change in subjective social status over the lifespan (e.g., MSSS adulthood minus MSSS childhood) in order to determine whether there is a unique relationship between change in subjective status and emotion perception that could inform whether emotion perception shapes, or is shaped by, social status. As shown in Supplementary Table S6, within our sample 21.6% reported no change in social status, 38.9% reported a decrease in social status, and 39.5% reported an increase in social status (M_∆MSSS_ = −0.07, SD = 2.01). When we examined preregistered H4 (Table 3), we found a negative effect of change in social status on emotion perception (*b* = −0.36, *t* = −2.18, *p* = 0.03, 95% CI = [−0.68, −0.04]), suggesting that an increase in subjective social status over time was associated with worse emotion perception abilities. However, this relationship would not survive a Bonferroni multiple comparison correction (alpha = 0.0167). Similar to the analyses described above, we did not observe significant effects of change in social status over the lifespan for the other subjective social status measures examined (see Supplementary Table S7).

**Table 3:** Change in subjective social status from childhood to adulthood negatively predicts emotion perception (preregistered H4) for individuals but not groups.

	Individual emotion perception task (GERT-S)	Group emotion perception task (EET)
Intercept	67.59***	1.03***
	[60.92, 74.25]	[0.73, 1.32]
Change in social status (MSSS)	−0.36*	−0.01
	[−0.68, −0.04]	[−0.02, 0.01]
Age	−0.06*	0.01***
	[−0.11, −0.01]	[0.005, 0.01]
Female	1.81**	−0.04
	[0.5, 3.12]	[−0.1, 0.02]
White	1.96*	−0.03
	[0.44, 3.47]	[−0.09, 0.04]
Democrat	1.85**	−0.04
	[0.54, 3.16]	[−0.1, 0.02]
Agreeableness	−0.29	0.02
	[−1.09, 0.51]	[−0.02, 0.05]
Sense of Power	−2.05**	0.01
	[−3.52, −0.58]	[−0.05, 0.08]

Note :^*t*^*p* < 0.10, * p <0.05, ** p < 0.01, *** p < 0.001; Table contains estimate, significance, [95% CI]. The difference score for subjective social status measure was calculated as subjective social status in adulthood minus subjective social status in childhood.

While our preregistered analyses suggest the potential for a negative relationship between change in social status and emotion perception, additional exploratory analyses examined whether change in social status provides additional explanatory power beyond current social status. This analysis is important given the potential overlap between current social status and change in social status (e.g., positive changes in social status will be associated with higher status in adulthood). In a hierarchical regression, we found that the change in social status does not provide additional explanatory power beyond that explained by current status (Δ*R*^*2*^ = 0.001, *F*_change_ (1, 1187) = 1.23, *p* > 0.05). In addition, including both current status and change in status in the model resulted in neither predictor being statistically significant (both *p* > 0.05), suggesting that the correlation between these variables (*r* = 0.47, *p* < 0.0001) is sufficiently large such that the effect of one predictor independent of the other is not statistically significant.

We next aimed to reduce the correlation between current social status and change in social status by subsampling the data to just those who place themselves in the middle of the MSSS in adulthood (on ladder rungs 4–6, n = 561). This middle range was selected because it has greater potential to include individuals who have moved up or down in status compared to individuals in the lower end of the MSSS (on ladder rungs 2–3, n = 339) who are more likely to have moved down and individuals in the upper end of the MSSS (on ladder rungs 7–8, n = 297) who are more likely to have moved up (see Table S6). Considering only this middle range reduced the correlation between current social status and change in social status to *r* = 0.24, *p* < 0.0001 (vs *r* = 0.47, *p* < 0.0001 in the full dataset). Moreover, while the relationship between current social status and emotion perception when controlling for confounds was no longer significant in the subsample (*b* = −0.55, *t* = −0.99, *p* = 0.32, 95% CI = [−1.64, 0.54]), the relationship between emotion perception and change in social status became significant (*b* = −0.69, *t* = −2.62, *p* = 0.01, 95% CI = [−1.21, −0.17]), and remained significant when controlling for current social status (change in MSSS: *b* = −0.67, *t* = −2.46, *p* = 0.01, 95% CI = [−1.2, −0.13]; current MSSS: *b* = −0.21, *t* = −0.38, *p* = 0.71, 95% CI = [−1.33, 0.9]; see Table S8). This suggests that when the correlation between current social status and change in social status was minimized, there was a negative relationship between change in social status and emotion perception that was independent of current social status. While we found this relationship among individuals in the middle of the MSSS, the findings did not replicate in the upper and lower levels of the MSSS (all *p* > 0.05), perhaps due to smaller sample sizes or more restricted ranges on one or both MSSS measures.

Last, we examined whether the change in social status (which is based on childhood social status reported retrospectively) was biased by current social status (e.g., whether individuals recall their childhood social status differently depending on their current social status). Given the correlation between current status and change in status, we created an orthogonalized indicator of change in social status (i.e., normalizing change in status relative to others with same current MSSS), where higher numbers indicate greater change in status relative to others who place themselves on the same ladder rung in adulthood. When examining this variable in relation to objective measures of current social status (income for self, income for others in household, education for self), we observed a positive relationship for one’s own income (*b* = 0.07, *t* = 4.78, *p* < 0.001, 95% CI = [0.04, 0.1]) and a negative relationship for education (*b* = −0.06, *t* = −2.86, *p* = 0.004, 95% CI = [−0.10, −0.02]), suggesting that current status does influence perceived change in status. That is, higher income was associated with reporting a greater increase/less of a decrease relative to others with the same status and greater education was associated with reporting less of an increase/more of a decrease in status relative to others with the same status.

Together, these results provided preliminary evidence of a relationship between change in social status and emotion perception. While our preregistered H4 suggests a negative relationship between change in social status and emotion perception, this relationship did not survive multiple comparison correction and exploratory analyses highlighted the overlap between current social status and change in social status. That is, the variables were moderately correlated such that controlling for current social status eliminated the effect of change in social status within the full sample and change in social status may be biased by current social status due to the retrospective nature of our measure. However, reducing the correlation between current social status and change in social status (i.e., by focusing on only those in the middle range of MSSS) revealed a negative relationship between change in social status and emotion perception within a subset of the sample. Future work will be needed to further explore this relationship.

### Negative relationship between subjective social status and emotion perception does not hold for emotion perception of groups

While the results above demonstrate a negative relationship between subjective social status and emotion perception of individuals, we found no evidence of a similar effect in the group emotion perception task. In our preregistered analyses, we examined group emotion perception in the context of precision scores (e.g., how close responses were to the true proportion), for which lower scores indicate more precise estimates (see Methods). Among precision scores, we did not observe significant effects of subjective social status (preregistered H2, H3, H4, see Tables 2, 3, S1, S4, and S7). However, for preregistered H2, we observed a significant negative correlation between objective social status (specifically, income for others in household) and group emotion perception (precision scores; *r*(1195) = −0.10, *p* = 0.001, 95% CI = [−0.15, −0.04]), suggesting that as objective social status increased, estimates in the group perception task became more precise. When controlling for known confounds of emotion perception (full model, preregistered H3), this negative relationship for income for others in household was significant (or marginal, depending on model; see Table 2 and S4). Similarly, in preregistered H4, we observed a marginal effect for the change in social status (SSC: *b* = −0.03, *t* = −1.87, *p* = 0.06, 95% CI = [−0.05, 0.001]), suggesting that participants whose social status increased from childhood to adulthood were more precise in their estimates for the group emotion perception task (i.e., closer to the true proportion of smiling faces). However, the overlap between current and change in social status, as described above, poses interpretive challenges for the group emotion perception analyses as well.

Exploratory analyses repeated the above analyses for measures of accuracy (instead of precision) in the group emotion perception task (see Supplementary Tables S9a and S9b). We observed a marginal positive correlation between accuracy in the group emotion perception task and subjective social status (SSC; *r*(1195) = 0.05, *p* = 0.09, 95% CI = [−0.01, 0.11], H2), again suggesting that higher subjective social status was associated with better emotion perception for groups. This effect was also marginal when controlling for known confounds of emotion perception (*b* = 0.76, *t* = 1.86, *p* = 0.06, 95% CI = [−0.04, 1.57], restricted model of H3). However, when also controlling for objective measures of social status, this effect was no longer marginal (*p* > 0.10; full model of H3; see Supplementary table S9a). In addition, there was a significant positive relationship between the change in social status from childhood to adulthood and accuracy in the group emotion perception task, (SSC; *b* = 0.91, *t* = 2.58, *p* = 0.01, 95% CI = [0.22, 1.6], H4), which parallels the marginal effect observed for the precision of estimates in the group task reported above.

### Robustness checks

The results reported above provide evidence for a negative relationship between social status and emotion perception of individuals but not individuals within a group. To further probe these relationships, we performed a number of exploratory analyses. With exception of the last analysis reported below, these exploratory analyses were limited to understanding the relationship between subjective social status (i.e., MSSS) and emotion perception for individuals (i.e., GERT-S), given the lack of significant results reported above for the other measures.

First, we examined whether the effects of social status on emotion perception of individuals extended to self-reported childhood social status. In an exploratory analysis, we examined the model described in preregistered H3 while using childhood MSSS instead of adulthood MSSS and found that the effects did not generalize to childhood social status (*b*_*childhood MSSS*_ = −0.04, *t* = −0.2, *p* > 0.05, 95% CI = [−0.37, 0.3]).

In addition, we tested for moderators of the effects using an exploratory analysis in which we added a gender-by-subjective-social-status interaction (MSSS) to the full model described in preregistered H3; that interaction was significant (*b* = 1.53, *t* = 4.3, *p* < 0.0001, 95% CI = [0.83, 2.23]), revealing a negative relationship between subjective social status and emotion perception of individuals for non-female participants (*b* = −1.28, *t* = −3.45, *p* < 0.001, 95% CI = [−2.01, −0.55]) but not female participants (*b* = 0.13, *t* = 0.39, *p* > 0.05, 95% CI = [−0.51, 0.76]). In addition, we tested for a race (white vs non-white)-by-subjective-social-status (MSSS) interaction using the full model described in preregistered H3, as well as a gender-by-subjective-social-status (MSSS) interaction in the model for preregistered H4. We did not observe any significant interactions, *p* > 0.05.

We also examined whether our conclusions would hold under an alternative scoring procedure for the individual emotion perception task. Using an alternative GERT-S score, in which accuracy was assigned based on the popularity of the response (see Methods), we re-ran our preregistered hypotheses. This alternative GERT-S score was highly correlated with the original GERT-S score, *r* = 0.95, *p* < 0.0001 and repeating our preregistered analyses with the alternative GERT-S score produced similar results to those reported above (see Supplementary Tables S10a and S10b).

Last, we considered the possibility that the observed effects were due to differential effort put forth by higher- and lower-status individuals in the incentive-compatible tasks (i.e., higher-status individuals perform worse in the tasks due to lack of effort or less need for bonus money). In an exploratory analysis, we examined whether performance in the control perception task, in which participants judged indoor and outdoor scenes, was related to social status. While we did not find an association between social status and emotion perception for individuals in groups (see preregistered H3), we observed a positive relationship between social status and precision scores in the control perception task (*b* = 0.03, *t* = 2.28, *p* = 0.02, 95% CI = [0.004, 0.05]), suggesting that higher social status individuals were less precise than lower status individuals in their perception of indoor and outdoor scenes (i.e., lower status individuals were closer to the true proportion). However, when adding performance in the control perception task to our models, the results reported above for both individual and group emotion perception held for hypothesis H3 – there was still a negative relationship for emotion perception of individuals (*b* = −0.54, *t* = −2.19, *p* = 0.03, 95% CI = [−1.02, −0.06]) but not groups (*p* > 0.05; see Supplementary table S11a). This suggests that differential sensitivity to the incentives could not explain our effects. This conclusion is also supported by the absence of status effects in the group perception task, which was similarly incentive-compatible. We also observed similar effects for hypothesis H4, with the exception that the marginally significant effect reported above between the change in SSC and group emotion perception became significant when also controlling for precision of estimates in the control perception task (see Supplementary table S11b).

### Despite robust relationships, overall effect sizes remain small

**Fig. 2:**
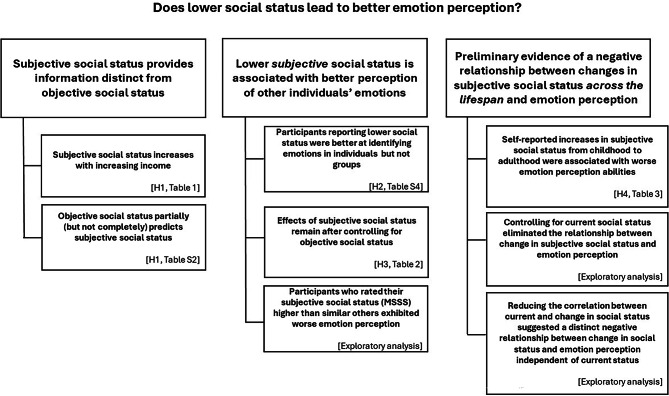
Summary of key findings and supporting analyses. Previous literature suggests differential effects of social status on emotion perception when including subjective and objective social status measures. Evidence from this study suggests that subjective and objective measures of social status are related but not equivalent. When including both subjective and objective measures of social status in models predicting emotion perception ability, there is an independent, significant negative effect of subjective social status. In addition, preliminary evidence suggests the change in subjective social status negatively predicts emotion perception ability, such that individuals who move up in social status (e.g., lower social status in childhood, higher social status in adulthood) exhibit worse emotion perception abilities while those who move down in social status exhibit better emotion perception. These findings are based on the MacArthur Subjective Social Status scale and the individual emotion perception task. Effects did not generalize to the group emotion perception task or other subjective measures of social status.

The analyses above demonstrated a significant negative relationship between social status and emotion perception which held even when controlling for confounding variables (see Fig. 2 for summary of results). Yet, it is also noteworthy that across all of our preregistered models, the overall variance explained by all factors included in the model was relatively small (e.g., 3 to 5% of total variance; see Supplementary Table S3). There was significant heterogeneity in people’s emotion perception abilities due to factors beyond social status and other measured confounds.

As an example of the consistent but small effect of social status, each additional step on the social status ladder was associated with a decrease in GERT-S accuracy by approximately half a percentage point (H3, restricted model, *b* = −0.45; full model *b* = −0.62). This pattern means that given the full range of the social status ladder, which consists of 10 rungs, we would expect about a 5–6% difference in accuracy between individuals at its extremes. This effect can be compared to that of other factors that are known to influence emotion perception, such as gender, race, and political party (H3 restricted model: parameter estimates range from 1.8–2.2). For comparison, individuals would have to be about 3–4 rungs different on the ladder scale for social status to have effects similar in magnitude to these other factors.

## Discussion

This study sought to understand the relationship between emotion perception abilities and social status. Using a battery of subjective and objective measures of social status, along with incentive-compatible emotion perception tasks that address criticisms of prior work, we found a negative relationship between individuals’ social status and empathic accuracy: specifically, those with lower social status were better at identifying emotions in other individuals. This effect was consistent across analyses and robustness checks but was relatively small and limited to a subset of subjective measures. These findings also align with previous literature showing small to moderate negative correlations between emotion perception and social status^7,8^.

This study extends our understanding of factors that drive individual differences in emotion perception by demonstrating that it is specifically the *subjective* aspects of social status that influence emotion perception ability. As may be expected, subjective and objective social status measures were closely related, suggesting individuals possess generally accurate representations of how their income and education position them within society. However, objective and subjective social status each provided unique explanatory power toward emotion perception abilities – and results indicate that the subjective aspects of social status specifically underlie effects on emotion perception. This pattern is consistent with studies that manipulate social status^8^; moreover, it highlights that emotion perception abilities may not depend on the absolute resources available to an individual but on their perception of those resources in relation to others^10^.

To further understand the relationship between emotion perception and social status, we considered changes in social status across the lifespan. We observed a negative relationship between the change in social status and emotion perception. However, exploratory analyses highlighted the need for further research, as current social status and change in social status were moderately correlated in our sample and we found that change in social status did not provide additional explanatory power beyond current status. However, when change in status and current status were only weakly correlated (i.e., within the subset of participants in the middle range of the MSSS), change in social status was predictive of emotion perception ability and current status was not. These results did not replicate in the subset of participants in the lower and upper ranges of the MSSS, which may be due to lack of power (i.e., small effect sizes, small sample sizes) or the restricted range in MSSS for each subset. This exploratory analysis is beyond our preregistration and should be replicated in future research. If found in a separate larger study designed to specifically address the correlation between current and change in status, it could provide evidence in support of the perspective that social status affects emotion perception ability (rather than being dependent on that ability) and could suggest that despite known benefits of social cognition for many aspects of life^2^, having better emotion perception abilities many not be a straightforward driver of social mobility.

This study highlights new boundary conditions for the relationship between social status and emotion perception. Unlike individual emotion perception, perceptions of the emotional status of people in a group were not predicted by social status, with the observed relationships only having marginal effects that were generally in the opposite direction (i.e., group perception was marginally better in higher-status individuals); such effects, if validated in future studies, would support conclusions that group and individual processes differ^33^. Another possible explanation could be that different processes are engaged by each of the tasks used, such that the GERT-S requires thinking about a single actor’s affective states while the EET requires a broader assessment of a group of people. We further note that the EET only included assessment of smiling and neutral faces. Thus, we cannot rule out the possibility that other group perception tasks (e.g., thinking about affective or mental states of individuals in a group, considering a wider variety of emotional expressions) could show similar effects to those observed here for individual emotion perception. Our exploratory analyses also suggested that the relationship between social status and individual emotion perception was not present for female participants. However, since this was an exploratory analysis, replicating this finding in a separate preregistered sample will be critical.

This study also includes various methodological advances in the study of social status and emotion perception. Whereas previous studies have used tasks such as Reading the Mind in the Eyes^7,8^, which includes static, eyes-only images paired with complex vocabulary, we opted for a more dynamic assessment of realistic emotional expressions within video clips; this approach helps minimize biases that benefit select economic and racial categories (e.g., based on IQ, education, cultural background^20,26^). In addition, we extend the study of social status effects to group emotion perception, using a task that assesses perception of ensembles of social and non-social images and we control for nonsocial perceptual abilities in our analyses. Our tasks were also incentive-compatible to encourage accurate performance. In addition, by examining a variety of social status measures, we found that the effects of social status were only present in select measures that best captured variability in subjective social status, focusing on the comparative nature of subjective social status.

While this study provides several findings and methodological advances, it also has several limitations. First, this study does not manipulate subjective social status (but see^8^ for an example of such manipulation), leaving the possibility that its effects were caused by another un-measured variable; however, we note that this concern is tempered by the independent measurement of social status outside of the task context (i.e., participants also indicated social status in pre-screening). In addition, this study was conducted using participants who may be more socially-minded and/or research-oriented than the general public due to the self-selective nature of online research platforms. Similarly, despite our goal of obtaining a sample that spans social status in the US – and the seeming successful achievement of that goal based on participants’ self-reports –the Prolific participant population could still differ in important ways from the full US population (e.g., it omits individuals who are at the extremes of the social status ladder). In addition, we asked participants to recall their childhood social status, which may be influenced by their experiences and current social status, as evidenced by our exploratory analyses. This highlights the importance of collecting longitudinal data in order to obtain a non-biased measurement of change in social status. Relatedly, current status and change in social status were correlated within our full sample, which made it challenging to ask whether change in social status is uniquely related to emotion perception. However, exploratory analyses within the subset of participants where current and change in social status were not correlated provide encouraging results. Last, we control for general perceptual abilities in our analyses, but did not include a non-emotional but social control task (e.g., identifying the proportion of women in an ensemble) that would allow us to rule out the possibility that lower status individuals pay greater attention to other people in general. These limitations provide direction for future research.

Overall, this study provides new insights into the relationship between social status and emotion perception. It highlights a robust, negative relationship between social status and emotion perception in individuals – and shows that subjective features of social status underlie this relationship. These findings suggest that emotion perception abilities may be context dependent. Moreover, it demonstrates that factors outside of social status also influence emotion perception. For example, our most detailed model that included demographics, known confounding factors, and objective and subjective measures of social status still only explained a small proportion of the variance in emotion perception abilities (i.e., about 5%). Emotion perception abilities are influenced but not determined by social status – and thus people may still improve their emotion perception abilities regardless of their social status. Future research could consider the advantages and disadvantages that come with varying levels of empathic accuracy for different levels of social status, as well as the mechanisms that give rise to and sustain such relationships.

## Methods

Study materials, deidentified data, and syntax used for analyses are available at: osf.io/gnc34. Data were collected and analyzed as outlined in our preregistration (10.17605/OSF.IO/NXT9E) and are summarized below. Any analyses that extended beyond the preregistration are explicitly described as “exploratory” hereafter. All procedures and methods were conducted in accordance with guidelines approved by the IRB at Duke University. Informed consent was obtained from all participants.

### Participants

Our pre-registered target sample size was 1250 participants. In previous work on this topic, Dietze & Knowles conducted power analyses which found that N = 450 would have a 95% power in detecting a small effect of a negative association between status and emotion perception accuracy^7^. To maximize statistical power for detecting the main effect of a linear association between ordinal class categories and emotion perception performance, we planned to recruit a sample of 1250 participants. Additionally, this larger sample size more closely matched the scale of the participant sample (N > 1000) in previous work that observed a positive relationship between emotion perception and objective measures of social status^19^. Lastly, the increased sample size accommodates follow-up moderation analyses and inspection of control variable distributions.

Data were collected from 1253 participants on Prolific in June 2022. Participants were required to be at least 18 years old, live in the United States, be fluent in English, have at least 98% of previous Prolific submissions approved, and have completed at least 10 previous Prolific submissions. In addition, participants had to have answered Prolific’s background data questions about Household Income, Employment Status, Personal Income, Current US State of Residence, and SES status. In order to ensure that our participant sample would include an approximately equal number of both high and low social status participants, we recruited participants based on their responses to the Prolific SES prescreening question (“Where would you put yourself on the socioeconomic ladder,” with response options from 1–10). Based on the number of eligible participants in each response option, we recruited a uniform distribution of participants across socioeconomic ladder rungs 2 through 8. Ladder rungs 1, 9, and 10 were excluded from recruitment due to the small number of participants available on Prolific in each of these categories. All analyses used the social status measures reported by participants during our data collection session (i.e., not the values recorded in Prolific demographics that were used for sample construction).

Data analyses include 1197 participants (age: M = 37.8, SD = 13.8, 50.1% female, 73.9% white, 50% Democrat). Participants were excluded from analyses if they did not complete the emotion perception tasks or the social status measures for self, if they provided inconsistent responses to the indices of social status (e.g., income for self > income for household), or if they indicated their current social status was outside of the targeted sample (i.e., ladder rungs 1, 9, or 10 on the MacArthur Subjective Social Status scale).

Participants were paid $4 for participation, with the opportunity to earn additional bonus money (up to $2) based on performance in the study.

### Procedures

Participants first completed two emotion perception tasks—one examining emotion perception ability for individuals (GERT-S) and another examining emotion perception ability for groups of individuals (EET). The order of these two tasks was randomized across participants. As part of the EET, participants also completed a control (non-social) perception task. Following these perception tasks, all participants completed a series of questionnaires asking about subjective and objective measures of social status, other control variables, and demographics.

### Measures

Participants completed the perception tasks described above. All were incentive-compatible such that participants could earn bonus money for accurate responses (e.g., identifying the correct emotion expressed in GERT-S, correct proportion of smiling faces/outdoor scenes in EET). For each task, participants were rank ordered based on performance and bonus payments were made on a sliding scale (e.g., the top 20% of participants received $1, the next 20% received $0.75, and so on).

In the individual emotion-perception task (Geneva Emotion Recognition Test, GERT-S^25^), participants viewed 42 short video clips of an actor displaying 1 of 14 emotions while speaking a sequence of meaningless syllables. Participants indicated the emotion expressed, with options including: pride, joy, amusement, pleasure, relief, interest, surprise, anxiety, fear, despair, sadness, disgust, irritation, and anger (see Figure 1a). Participants viewed the video clips in a randomized order, with actors varying across video (5 male and 5 female white actors, ranging in age from 25–57 years old) and each emotion represented a total of three times. The GERT-S was selected as the individual perception task because it is an established task^25^ whose stimuli were selected for characteristics relevant to emotion perception (e.g., intensity, believability, and others’ ability to recognize the emotions). The stimuli offer dynamic visual and audio cues from actors who engaged with a professional, off-camera director based on real-life scenarios in order to ensure maximal emotion induction and high authenticity of the emotional expressions. Moreover, the GERT-S has been shown to be a reliable (Cronbach’s alpha = 0.8 or higher) unidimensional test of emotion recognition that is positively correlated with other emotional recognition tasks, such as the Situational Test of Emotional Understanding (STEU^34^) and the Diagnostic Analysis of Nonverbal Accuracy (DANVA^35^).

In the group emotion-perception task (Ensemble Emotion Task, EET^27^), participants viewed brief displays (1 s) of an ensemble of 12 faces and estimated the proportion of smiling individuals. Faces within each ensemble were matched on gender (6 male, 6 female) and race (6 Black, 6 White) and consisted of either neutral faces or closed-mouth smiling faces (taken from the Chicago Face Database^36^). The proportion of smiling faces varied from 0 to 100% in increments of 25%, for a total of 5 unique proportions. Other facial features (e.g., attractiveness, trustworthiness) were randomly drawn from the CFD using the same procedures as Khaw et al., 2021. As part of the EET, participants also completed a control perception task^27^, in which they viewed ensembles of indoor and outdoor scenes and estimated the proportion of outdoor scenes. (see Fig. 1b).

Subjective measures of social status included the MacArthur Subjective Social Status (MSSS^29,30^), Subjective Social Class (SSC^32^) and Subjective SES Scale (SSS^31^). These measures were selected due to the potential to capture different aspects of subjective social status. The MSSS measure is a continuous measure that asks participants to think about their standing in a social hierarchy^37^. Participants were shown a ladder in which the highest rungs represent the people who are best off (e.g., most money, education, career respect) and the lowest rungs those who are worst off and then indicated the rung that best represents where they are on that ladder (from 1 to 10). The SSC measure asks participants to indicate in which of five subjective social classes they belong: poor, working class, middle class, upper middle class, and upper class. This framework has been shown to also capture subjective features of social class associated with lifestyle (e.g., dress, language, etiquette, values)^38^. The SSS measure is more directly related to having needs met. Participants are asked to rate a series of statements on a 7-point scale, from strongly disagree to strongly agree, such as “I have enough money to buy things I want” and “I don’t need to worry too much about paying my bills.” For each of these measures, participants were asked to self-report both their current and childhood social status.

Objective measures of social status included income (for total household, self, others in household, and family/childhood) and educational attainment (for self and parents). Income was collected using predetermined deciles corresponding to the US income distribution in 2021, such that approximately 10% of the distribution falls within each of the 10 categories (e.g., ranging from less than 15,000 to greater than $200,000). Education was operationalized as the highest achieved education level for the participant and up to two parents.

In addition, we collected information related to known confounds of emotion perception. This included demographics (e.g., age, gender, race/ethnicity, political affiliation) and scale measures of agreeableness (short form^39^), and sense of power^40^. Each of these variables have been previously linked to social cognitive abilities^19,41–45^^.^.

### Data preprocessing/transformations

For our analyses, we created composite scores and dummy variables for select measures. For the individual emotion perception task (GERT-S), we calculated accuracy as the proportion of trials on which the emotion of the speaker was correctly identified. For the group emotion perception task (EET) and the control scene perception task, we examined the precision of the estimates provided by calculating the average absolute value of the difference between each participant’s judgment and the true proportion of smiling faces (or outdoor scenes). For this measure, lower scores indicate better performance (i.e., more precise estimates). In addition, an accuracy score for the EET was calculated as the proportion of trials on which the true proportion of smiling faces was correctly identified.

Composite scores were created for scale measures by taking an average score for each measure. Additionally, we calculated the change in social status from childhood to adulthood for each measure (e.g., MSSS for adult minus MSSS for childhood). Demographic variables were dummy coded for gender (female vs not), race/ethnicity (non-Hispanic White vs not), and political affiliation (Democrat vs not). Age was calculated as current year minus birth year. Income for others in the household was calculated as household income minus income for self.

To further probe our findings, we also created alternative scoring measures for the GERT-S and the MSSS scale. To address potential confusability of emotions within the GERT-S (e.g., joy may be confused with pleasure), we calculated an alternative accuracy measure that incorporates the distribution of responses to each video (i.e., a graded accuracy score in which popular response options receive more weight; see Supplementary Table S11). For the alternative scoring of the MSSS, we took each participant’s MSSS score and subtracted the mean MSSS score of the social class category they selected in the SSC measure. This normalization allowed us to examine the extent to which participants used similar scales in their subjective assessments on the MSSS relative to those who choose the same subjective social class in the SSC measure; for example, two individuals might both describe themselves as “middle class”, but one rates themselves as a 7 on the MSSS measure and the other rates themselves a 4.

In addition, exploratory analyses revealed a moderate correlation between current status and change in social status (MSSS). To orthogonalize these measures, we z-scored the change in social status using the mean change in social status for each level of current social status. This provides a measure that corrects for the typical change associated with a given current status; for example, a raw change score of 4 ladder steps would have a higher z-score if it were from step 1 to step 5 (i.e., relatively rare) than from step 4 to step 8 (i.e., relatively common).

### Hypotheses and data analysis

We tested four preregistered hypotheses surrounding the relationship between social status and emotion perception ability. First, we hypothesized that subjective social status measures would be grounded in, but not equivalent to, objective measures of social class *(H1: Subjective social status is at least partially explained by objective socioeconomic indicators.)*. Second, we hypothesized that the correlational effect reported in the literature would be present in our data *(H2: Higher social status is associated with lower emotion perception accuracy)*. Third, we hypothesized that subjective social status would have an effect on emotion perception even when controlling for demographics, other known confounds, and objective measures of social status *(H3: Self-categorized social status is a significant predictor of emotion perception accuracy, controlling for objective measures of social status and other known confounds of emotion perception).* Last, we hypothesized that the change in social status over the lifetime would be a significant predictor of emotion perception ability *(H4: Changes in social status from childhood to adulthood is significantly related to emotion perception accuracy).*

All analyses were conducted in SAS 9.4. We test these hypotheses using correlations (H2) and multiple linear regression (H1, H3, H4). Further details about predictors included in each model are available in the results section and our preregistration (10.17605/OSF.IO/NXT9E). While we preregistered the use of household income as an objective measure of social status, the analyses reported here separate income for self and income for others in the household. This minimizes the redundancy between income for self and overall income for household (i.e., income for self is part of household income for multi-person households and equivalent to household income for single-person households).

In addition to our preregistered analyses, we conducted follow-up exploratory analyses in order to examine moderators, boundary conditions, and robustness checks of the observed effect. We also explored the extent to which subjective social status drives the observed effects. These exploratory analyses were limited to the examination of the effect of the MSSS score, as we did not find significant effects for the SSC or SSS measures in our preregistered analyses.

## Supplementary Information

Supplementary Information.

## Data Availability

Study materials, deidentified data, and syntax used for analyses are available at: osf.io/gnc34. Data were collected and analyzed as outlined in our preregistration (10.17605/OSF.IO/NXT9E).

## References

[1] Frith, C. D. & Frith, U. Social Cognition in Humans. *Curr. Biol.*10.1016/j.cub.2007.05.068 (2007). 17714666 10.1016/j.cub.2007.05.068

[2] Frith, C. D. & Frith, U. Mechanisms of social cognition. *Annu. Rev. Psychol.* **63**, 287–313 (2012). 21838544 10.1146/annurev-psych-120710-100449

[3] Muscatell, K. A. et al. Social status modulates neural activity in the mentalizing network. *Neuroimage* **60**, 1771–1777 (2012). 22289808 10.1016/j.neuroimage.2012.01.080 PMC3909703

[4] Dietze, P. & Knowles, E. D. Social Class and the Motivational Relevance of Other Human Beings: Evidence From Visual Attention. *Psychol. Sci.* **27**, 1517–1527 (2016). 27698091 10.1177/0956797616667721

[5] Piff, P. K. & Moskowitz, J. P. The class—Compassion gap: How socioeconomic factors influence compassion. In *The Oxford handbook of compassion science* (eds Seppälä, E. M. et al.) 317–330 (Oxford University Press, 2017).

[6] Talaifar, S., Buhrmester, M. D., Ayduk, Ö. & Swann, W. B. Asymmetries in Mutual Understanding: People With Low Status, Power, and Self-Esteem Understand Better Than They Are Understood. *Perspect. Psychol. Sci.* **16**, 338–357 (2021). 33074793 10.1177/1745691620958003

[7] Dietze, P. & Knowles, E. D. Social Class Predicts Emotion Perception and Perspective-Taking Performance in Adults. *Pers. Soc. Psychol. Bull.* **47**, 42–56 (2021). 32336209 10.1177/0146167220914116

[8] Kraus, M. W., Côté, S. & Keltner, D. Social Class, Contextualism, and Empathic Accuracy. *Psychol. Sci.* **21**, 1716–1723 (2010). 20974714 10.1177/0956797610387613

[9] Kraus, M. W., Piff, P. K. & Keltner, D. Social class as culture: The convergence of resources and rank in the social realm. *Curr. Dir. Psychol. Sci.* **20**, 246–250 (2011).

[10] Kraus, M. W., Piff, P. K., Mendoza-Denton, R., Rheinschmidt, M. L. & Keltner, D. Social class, solipsism, and contextualism: How the rich are different from the poor. *Psychol. Rev.* **119**, 546–572 (2012). 22775498 10.1037/a0028756

[11] Gallo, L. C., Matthews, K. A., Bogart, L. M. & Vranceanu, A. M. Socioeconomic status, resources, psychological experiences, and emotional responses: A test of the reserve capacity model. *J. Pers. Soc. Psychol.* **88**, 386–399 (2005). 15841865 10.1037/0022-3514.88.2.386

[12] Dittmann, A. G., Stephens, N. M. & Townsend, S. S. M. Achievement Is Not Class-Neutral: Working Together Benefits People From Working-Class Contexts. *J. Pers. Soc. Psychol.* **119**, 517–539 (2020). 32378921 10.1037/pspa0000194

[13] Batson, C. D., Lishner, D. A. & Stocks, E. L. The Empathy-Altruism Hypothesis. In *The Oxford Handbook of Prosocial Behavior* (eds Schroeder, D. A. & Graziano, W. G.) 259–281 (Oxford University Press, 2015).

[14] Piff, P. K., Kraus, M. W., Côté, S., Cheng, B. H. & Keltner, D. Having Less, Giving More: The Influence of Social Class on Prosocial Behavior. *J. Pers. Soc. Psychol.* **99**, 771–784 (2010). 20649364 10.1037/a0020092

[15] Wei, B., Zhang, X., Cui, D. & Li, Y. Linking objective and subjective social status to altruistic sharing in China: The role of empathy. *Curr. Psychol.* **42**, 27401–27414 (2023).

[16] Esping-Andersen, G. & Cimentada, J. Ability and mobility: The relative influence of skills and social origin on social mobility. *Soc. Sci. Res.* **75**, 13–31 (2018). 30080485 10.1016/j.ssresearch.2018.06.003

[17] Boyatzis, R., Rochford, K. & Cavanagh, K. V. Emotional intelligence competencies in engineer’s effectiveness and engagement. *Career Dev. Int.*10.1108/CDI-08-2016-0136 (2017).

[18] Rode, J. C., Arthaud-Day, M., Ramaswami, A. & Howes, S. A time-lagged study of emotional intelligence and salary. *J. Vocat. Behav.* **101**, 77–89 (2017).

[19] Deveney, C. M. et al. How generalizable is the inverse relationship between social class and emotion perception?. *PLoS ONE*10.1371/journal.pone.0205949 (2018). 30339671 10.1371/journal.pone.0205949 PMC6195285

[20] Dodell-Feder, D., Ressler, K. J. & Germine, L. T. Social cognition or social class and culture? on the interpretation of differences in social cognitive performance. *Psychol. Med.* **50**, 133–145 (2020). 30616706 10.1017/S003329171800404X

[21] Lee, S. et al. Reading the Mind in the Eyes: A Population-Based Study of Social Cognition in Older Adults. *Am. J. Geriatr. Psychiatr.* **29**, 634–642 (2021). 10.1016/j.jagp.2020.11.009 PMC8166961 33293250

[22] Andrei, F., Mancini, G., Mazzoni, E., Russo, P. M. & Baldaro, B. Social status and its link with personality dimensions, trait emotional intelligence, and scholastic achievement in children and early adolescents. *Learn. Individ. Differ.* **42**, 97–105 (2015).

[23] Tracy, J. L. & Robins, R. W. The Automaticity of Emotion Recognition. *Emotion* **8**, 81–95 (2008). 18266518 10.1037/1528-3542.8.1.81

[24] Haberman, J. & Whitney, D. Seeing the Mean: Ensemble Coding for Sets of Faces. *J. Exp. Psychol. Hum. Percept. Perform.* **35**, 718–734 (2009). 19485687 10.1037/a0013899 PMC2696629

[25] Schlegel, K. & Scherer, K. R. Introducing a short version of the Geneva Emotion Recognition Test (GERT-S): Psychometric properties and construct validation. *Behav. Res. Methods* **48**, 1383–1392 (2016). 26416137 10.3758/s13428-015-0646-4

[26] Baker, C. A., Peterson, E., Pulos, S. & Kirkland, R. A. Eyes and IQ: A meta-analysis of the relationship between intelligence and ‘Reading the Mind in the Eyes’. *Intelligence* **44**, 78–92 (2014).

[27] Khaw, M. W., Kranton, R. & Huettel, S. Oversampling of minority categories drives misperceptions of group compositions. *Cognition*10.1016/j.cognition.2021.104756 (2021). 33971528 10.1016/j.cognition.2021.104756 PMC8628853

[28] Smith, V. L. Microeconomic Systems as an Experimental Science. *Am. Econ. Rev.* **72**, 923–955 (1982).

[29] Adler, N. E., Epel, E. S., Castellazzo, G. & Ickovics, J. R. Relationship of subjective and objective social status with psychological and physiological functioning: Preliminary data in healthy white women. *Health Psychol.* **19**, 586–592 (2000). 11129362 10.1037//0278-6133.19.6.586

[30] Goodman, E. et al. Adolescents’ Perceptions of Social Status: Development and Evaluation of a New Indicator. *Pediatrics* **108**, e31 (2001). 11483841 10.1542/peds.108.2.e31

[31] Mittal, C. & Griskevicius, V. Sense of Control Under Uncertainty Depends on People’s Childhood Environment: A Life History Theory Approach. *J. Pers. Soc. Psychol.* **107**, 621–637 (2014). 25133717 10.1037/a0037398

[32] Jackman, M. R. & Jackman, R. W. *Class Awareness in the United States* (Univ of California Press, 1983).

[33] Milch, K. F., Weber, E. U., Appelt, K. C., Handgraaf, M. J. J. & Krantz, D. H. From individual preference construction to group decisions: Framing effects and group processes. *Organ. Behav. Hum. Decis. Process* **108**, 242–255 (2009).

[34] MacCann, C. & Roberts, R. D. New Paradigms for Assessing Emotional Intelligence: Theory and Data. *Emotion*10.1037/a0012746 (2008). 18729584 10.1037/a0012746

[35] Nowicki, S. & Duke, M. P. Individual differences in the nonverbal communication of affect: The diagnostic analysis of nonverbal accuracy scale. *J. Nonverbal Behav.*10.1007/BF02169077 (1994).

[36] Ma, D. S., Correll, J. & Wittenbrink, B. The Chicago face database: A free stimulus set of faces and norming data. *Behav. Res. Methods* **47**, 1122–1135 (2015). 25582810 10.3758/s13428-014-0532-5

[37] Galvan, M. J., Payne, B. K., Hannay, J., Georgeson, A. R. & Muscatell, K. A. What Does the MacArthur Scale of Subjective Social Status Measure? Separating Economic Circumstances and Social Status to Predict Health. *Annals of Behavioral Medicine* **57**, (2023). 10.1093/abm/kaad054 37742041

[38] Diemer, M. A., Mistry, R. S., Wadsworth, M. E., López, I. & Reimers, F. Best practices in conceptualizing and measuring social class in psychological research. *Anal. Soc. Iss. Public Policy*10.1111/asap.12001 (2013).

[39] Gosling, S. D., Rentfrow, P. J. & Swann, W. B. A very brief measure of the Big-Five personality domains. *J. Res. Pers.* **37**, 504–528 (2003).

[40] Anderson, C., John, O. P. & Keltner, D. The Personal Sense of Power. *J. Pers.*10.1111/j.1467-6494.2011.00734.x (2012). 21446947 10.1111/j.1467-6494.2011.00734.x

[41] Barrett, L. F., Lane, R. D., Sechrest, L. & Schwartz, G. E. Sex Differences in Emotional Awareness. *Pers. Soc. Psychol. Bull.* **26**, 1027–1035 (2000).

[42] Halberstadt, J., Ruffman, T., Murray, J., Taumoepeau, M. & Ryan, M. Emotion Perception Explains Age-Related Differences in the Perception of Social Gaffes. *Psychol. Aging.* **26**, 133–136 (2011). 21280951 10.1037/a0021366

[43] Joseph, D. L. & Newman, D. A. Emotional Intelligence: An Integrative Meta-Analysis and Cascading Model. *J. Appl. Psychol.* **95**, 54–78 (2010). 20085406 10.1037/a0017286

[44] Vigil, J. M. Political leanings vary with facial expression processing and psychosocial functioning. *Group Process. Intergroup Relat.* **13**, 547–558 (2010).

[45] Blader, S. L., Shirako, A. & Chen, Y. R. Looking Out From the Top: Differential Effects of Status and Power on Perspective Taking. *Pers. Soc. Psychol. Bull.*10.1177/0146167216636628 (2016). 27036500 10.1177/0146167216636628

