# Supplementary material for: Higher self-assessed subjective social status is associated with worse perception of others’ emotions
Source: Sci Rep. 2025 May 17;15:17188. doi: 10.1038/s41598-025-01493-2 (PMC12085666; doi:10.1038/s41598-025-01493-2)
Supplement: Supplementary file 1 — Supplementary Information. [file 41598_2025_1493_MOESM1_ESM.docx]

Supplementary Materials for

**Higher self-assessed subjective social status is associated with worse perception of others’ emotions**

[author information removed for review]

**This PDF file includes:**

Section S1. Differences in task performance by demographic.

Section S2. Positive correlation between income for others in the household and emotion perception.

Table S1. Correlations between measures of social status and emotion perception ability. (including preregistered H2)

Table S2. Subjective social status is partially explained by objective measures of social status (preregistered H1)

Table S3. Overall variance explained (R^2^) in preregistered regression analyses.

Table S4. Effect of subjective social status (SSC & SSS) on emotion perception of individuals and groups. (preregistered H3)

Table S5. Overestimation of subjective social status (MSSS relative to SSC) negatively predicts emotion perception for individuals (exploratory analysis of H3).

Table S6. Change in subjective social status (MSSS) by current social status

Table S7. Effect of change in subjective social status (SSC & SSS) on emotion perception of individuals and groups. (preregistered H4)

Table S8. Effect of change in subjective social status (MSSS) on emotion perception of individuals for subset of participants in the middle of the MSSS ladder (rungs 4-6)

Table S9a. Effect of subjective social status on accuracy in the group emotion perception task (exploratory analysis of H3)

Table S9b. Effect of the change in subjective social status from childhood to adulthood on accuracy in the group emotion perception task (exploratory analysis of H4)

Table S10a. Effect of subjective social status on the alternative scoring of the individual emotion perception task (exploratory analysis of H3)

Table S10b. Effect of subjective social status on the alternative scoring of the individual emotion perception task (exploratory analysis of H4)

Table S11a. Effect of subjective social status controlling for performance in the nonsocial perception task (exploratory analysis of H3)

Table S11b. Effect of the change in subjective social status controlling for performance in the nonsocial perception task (exploratory analysis of H4)

Table S12: Distribution of responses used to create alternative GERT score.

Section S1. Differences in task performance by demographic.

In the individual emotion-perception task (GERT-S), we observed differences in performance by demographic, with female participants more accurate (60.7% accurate (95% CI = [59.8, 61.6]) than non-female participants (58.7% accurate, 95% CI = [57.8, 59.7]), *t*(1195) = -2.98, *p* = .003, white participants more accurate (60.2% accurate, 95% CI = [59.4, 60.9]) than non-white participants (58.4% accurate, 95% CI = [57.1, 59.7]), *t*(1195) = -2.29, *p* = .02, and Democrats more accurate (60.7% accurate (95% CI = [59.9, 61.6]) than non-Democrats (58.7% accurate, 95% CI = [57.7, 59.6]), *t*(1195) = -3.1, *p* = .002.

In the group emotion-perception task, we did not observe differences by gender or race for either accuracy or precision. However, we did observe a difference by political affiliation, such that Democrats (M = 1.34, 95% CI = [1.30, 1.38]) were more precise (i.e., over- or under-estimated the number of smiling faces to a lesser extent) than non-Democrats (M = 1.4, 95% CI = [1.35, 1.44]), *t*(1171.4) = 1.97, *p* = .05.

Section S2. Positive correlation between income for others in the household and emotion perception.

To further understand the positive correlation between emotion perception and income for others in the household, we considered whether the relationship may have resulted from a difference between single-income households (for which income for others would be equal to zero) and multi-income households (with non-zero income for others). For multi-income households, the positive correlation between income for others in the household and emotion perception was not significant (*r*(664) = 0.06, *p* > .05, 95% CI = [-0.02, 0.13]). In addition, t-tests revealed that individuals in multi-income households were better at emotion perception (60.4% accurate) than individuals in single-income households (58.8% accurate, *t*(1195) = -2.38, p = 0.02). Together, these results suggest a distinction between single- and multi-income households rather than a specific relationship between emotion perception and others’ income.

Table S1. Correlations between measures of social status and emotion perception ability. (including preregistered H2)

|  | **MSSS** | **SSC** | **SSS** | **Education for self** | **Income for self** | **Income for others in household** | **Total household income** | **GERT accuracy** | **EET accuracy** |
| --- | --- | --- | --- | --- | --- | --- | --- | --- | --- |
| **SSC** | 0.81 *** |  |  |  |  |  |  |  |  |
|  | [0.79, 0.83] |  |  |  |  |  |  |  |  |
| **SSS** | 0.7 *** | 0.69 *** |  |  |  |  |  |  |  |
|  | [0.67, 0.72] | [0.66, 0.72] |  |  |  |  |  |  |  |
| **Education for self** | 0.44 *** | 0.42 *** | 0.34 *** |  |  |  |  |  |  |
|  | [0.39, 0.48] | [0.37, 0.47] | [0.29, 0.39] |  |  |  |  |  |  |
| **Income for self** | 0.53 *** | 0.52 *** | 0.49 *** | 0.5 *** |  |  |  |  |  |
|  | [0.49, 0.57] | [0.48, 0.56] | [0.44, 0.53] | [0.46, 0.54] |  |  |  |  |  |
| **Income for others in household** | 0.22 *** | 0.29 *** | 0.17 *** | -0.07 * | -0.35 *** |  |  |  |  |
|  | [0.16, 0.27] | [0.24, 0.35] | [0.12, 0.23] | [-0.13, -0.01] | [-0.40, -0.30] |  |  |  |  |
| **Total household income** | 0.67 *** | 0.72 *** | 0.59 *** | 0.4 *** | 0.61 *** | 0.52 *** |  |  |  |
|  | [0.63, 0.70] | [0.69, 0.75] | [0.55, 0.63] | [0.35, 0.44] | [0.58, 0.65] | [0.48, 0.56] |  |  |  |
| **GERT accuracy** | -0.06 * | -0.02 | -0.03 | 0.02 | -0.09 ** | 0.08 ** | -0.02 |  |  |
|  | [-0.12, -0.01] | [-0.08, 0.04] | [-0.08, 0.03] | [-0.04, 0.08] | [-0.15, -0.04] | [0.02, 0.13] | [-0.08, 0.04] |  |  |
| **EET accuracy** | 0.03 | 0.05 t | -0.02 | -0.02 | -0.02 | 0.08 ** | 0.05 | 0.09 ** |  |
|  | [-0.02, 0.09] | [-0.01, 0.11] | [-0.08, 0.03] | [-0.07, 0.04] | [-0.08, 0.04] | [0.02, 0.13] | [-0.01, 0.10] | [0.04, 0.15] |  |
| **EET precision** | -0.01 | -0.02 | 0.01 | 0.01 | 0.03 | -0.1 *** | -0.05 t | -0.2 *** | -0.67 *** |
|  | [-0.07, 0.05] | [-0.08, 0.03] | [-0.05, 0.07] | [-0.04, 0.07] | [-0.02, 0.09] | [-0.15, -0.04] | [-0.11, 0.01] | [-0.25, -0.14] | [-0.70, -0.64] |

*Note: ^t^p < .10, * p < .05, ** p < .01, *** p < .001, Table contains correlation coefficients, significance, and 95%CI.*

Table S2. Subjective social status is partially explained by objective measures of social status (preregistered H1)

|  | MSSS | SSC | SSS |
| --- | --- | --- | --- |
| **Intercept** | 1.75***  [1.48, 2.01] | 1.02***  [0.9, 1.14] | 1.32***  [1.05, 1.59] |
| **Income for self** | 0.47***  [0.43, 0.5] | 0.24***  [0.22, 0.26] | 0.42***  [0.38, 0.46] |
| **Income for others in household** | 0.36***  [0.33, 0.4] | 0.21***  [0.2, 0.23] | 0.29***  [0.26, 0.33] |
| **Education for self** | 0.19***  [0.13, 0.24] | 0.08***  [0.05, 0.1] | 0.08**  [0.02, 0.13] |

*Note: * p < .05, ** p < .01, *** p < .001, Table contains estimate, significance, [95% CI]*

Table S3. Overall variance explained (R^2^) in preregistered regression analyses.

| **Model description** | **R^2^** | |
| --- | --- | --- |
| Preregistered H1 |  | |
| Objective social status predicting MSSS | 0.49 | |
| Objective social status predicting SSC | 0.55 | |
| Objective social status predicting SSS | 0.38 | |
| Preregistered H3 | GERT | EET |
| Effect of MSSS on emotion perception (restricted model) | 0.03 | 0.04 |
| Effect of MSSS on emotion perception (full model) | 0.05 | 0.04 |
| Effect of SSC on emotion perception (restricted model) | 0.03 | 0.04 |
| Effect of SSC on emotion perception (full model) | 0.04 | 0.04 |
| Effect of SSS on emotion perception (restricted model) | 0.03 | 0.04 |
| Effect of SSS on emotion perception (full model) | 0.04 | 0.05 |
| Preregistered H4 | GERT | EET |
| Effect of the change in MSSS on emotion perception | 0.03 | 0.04 |
| Effect of the change in SSC on emotion perception | 0.03 | 0.04 |
| Effect of the change in SSS on emotion perception | 0.03 | 0.04 |

Note: Table contains the proportion of variance explained by the overall model tested for each preregistered regression analysis.

Table S4. Effect of subjective social status (SSC & SSS) on emotion perception of individuals and groups. (preregistered H3)

|  | **SSC** | | | | **SSS** | | | |
| --- | --- | --- | --- | --- | --- | --- | --- | --- |
|  | **Individual**  **emotion perception task (GERT-S)** | | **Group**  **emotion perception task (EET)** | | **Individual**  **emotion perception task (GERT-S)** | | **Group**  **emotion perception task (EET)** | |
|  | **Restricted Model** | **Full Model** | **Restricted Model** | **Full Model** | **Restricted Model** | **Full Model** | **Restricted Model** | **Full Model** |
| **Intercept** | 68.75*** | 67.49*** | 1.07*** | 1.07*** | 68.42*** | 67.16*** | 1.03*** | 1.06*** |
|  | [61.73, 75.76] | [60.42, 74.56] | [0.76, 1.38] | [0.76, 1.38] | [61.56, 75.28] | [60.18, 74.13] | [0.73, 1.33] | [0.75, 1.37] |
| **Subjective social status measure** | -0.39 | -0.35 | -0.01 | 0.01 | -0.2 | -0.08 | -0.001 | 0.01 |
|  | [-1.12, 0.33] | [-1.42, 0.71] | [-0.05, 0.02] | [-0.04, 0.05] | [-0.58, 0.18] | [-0.56, 0.39] | [-0.02, 0.02] | [-0.01, 0.03] |
| **Age** | -0.07** | -0.05* | 0.01*** | 0.01*** | -0.06* | -0.05^t^ | 0.01*** | 0.01*** |
|  | [-0.12, -0.02] | [-0.1, -0.0002] | [0.01, 0.01] | [0.005, 0.01] | [-0.11, -0.02] | [-0.1, 0.0004] | [0.01, 0.01] | [0.005, 0.01] |
| **Female** | 1.79** | 1.24^t^ | -0.04 | -0.03 | 1.79** | 1.24^t^ | -0.04 | -0.03 |
|  | [0.48, 3.11] | [-0.11, 2.58] | [-0.1, 0.02] | [-0.09, 0.03] | [0.48, 3.11] | [-0.11, 2.59] | [-0.1, 0.02] | [-0.09, 0.03] |
| **White** | 2.13** | 2.1** | -0.02 | -0.02 | 2.14** | 2.1** | -0.02 | -0.02 |
|  | [0.62, 3.65] | [0.59, 3.61] | [-0.09, 0.04] | [-0.09, 0.05] | [0.62, 3.65] | [0.59, 3.61] | [-0.09, 0.04] | [-0.09, 0.05] |
| **Democrat** | 1.85** | 1.79** | -0.04 | -0.04 | 1.81** | 1.77** | -0.04 | -0.04 |
|  | [0.54, 3.16] | [0.48, 3.11] | [-0.09, 0.02] | [-0.1, 0.02] | [0.51, 3.12] | [0.45, 3.09] | [-0.1, 0.02] | [-0.1, 0.02] |
| **Agreeableness** | -0.3 | -0.41 | 0.02 | 0.02 | -0.32 | -0.41 | 0.02 | 0.02 |
|  | [-1.1, 0.5] | [-1.21, 0.39] | [-0.02, 0.05] | [-0.02, 0.05] | [-1.12, 0.49] | [-1.21, 0.39] | [-0.02, 0.05] | [-0.02, 0.05] |
| **Sense of Power** | -2.06** | -2.11** | 0.01 | 0.01 | -2.04** | -2.09** | 0.01 | 0.01 |
|  | [-3.53, -0.59] | [-3.58, -0.65] | [-0.05, 0.08] | [-0.05, 0.08] | [-3.51, -0.57] | [-3.56, -0.63] | [-0.05, 0.08] | [-0.05, 0.08] |
| **Income for self** |  | -0.45* |  | -0.01 |  | -0.50* |  | -0.01 |
|  |  | [-0.87, -0.02] |  | [-0.03, 0.01] |  | [-0.89, -0.10] |  | [-0.03, 0.01] |
| **Income for others in household** |  | 0.25 |  | -0.02^t^ |  | 0.2 |  | -0.02* |
|  |  | [-0.14, 0.63] |  | [-0.03, 0.001] |  | [-0.15, 0.54] |  | [-0.03, -0.002] |
| **Education for self** |  | 0.5* |  | -0.002 |  | 0.48* |  | -0.002 |
|  |  | [0.03, 0.98] |  | [-0.02, 0.02] |  | [0.01, 0.95] |  | [-0.02, 0.02] |

*Note: ^t^p < .10, * p < .05, ** p < .01, *** p < .001; Table contains estimate, significance, [95% CI]. The effect of subjective social status on emotion perception was tested using separate models, each containing one subjective social status measure. Column headings indicate which subjective social status measure was included in the model.*

Table S5. Overestimation of subjective social status (MSSS relative to SSC) negatively predicts emotion perception for individuals (exploratory analysis of H3).

|  | **Restricted Model** | **Full Model** |
| --- | --- | --- |
| **Intercept** | 67.36***  [60.71, 74.02] | 66.4***  [59.47, 73.32] |
| **Overestimation of MSSS** | -0.94**  [-1.55, -0.32] | -0.85**  [-1.47, -0.23] |
| **Age** | -0.06*  [-0.11, -0.01] | -0.05*  [-0.1, -0.0003] |
| **Female** | 1.87**  [0.56, 3.18] | 1.33^t^  [-0.01, 2.68] |
| **White** | 2.08**  [0.57, 3.59] | 2.07**  [0.56, 3.57] |
| **Democrat** | 1.74**  [0.44, 3.05] | 1.68*  [0.37, 3] |
| **Agreeableness** | -0.22  [-1.02, 0.58] | -0.33  [-1.13, 0.47] |
| **Sense of Power** | -2.06**  [-3.53, -0.6] | -2.12**  [-3.58, -0.66] |
| **Income for self** |  | -0.47**  [-0.81, -0.13] |
| **Income for others in household** |  | 0.17  [-0.14, 0.49] |
| **Education for self** |  | 0.53*  [0.06, 0.99] |

*Note:  ^t^p < .10, * p < .05, ** p < .01, *** p < .001; Table contains estimate, significance, [95% CI].*

*.*

Table S6. Change in subjective social status (MSSS) by current social status

|  | **Current MSSS** | | | | | | | |
| --- | --- | --- | --- | --- | --- | --- | --- | --- |
|  | Full sample | Lower | | Middle | | | Upper | |
| **Change in MSSS** | 2 through 8 | 2 | 3 | 4 | 5 | 6 | 7 | 8 |
| **-7** | 0.17 | 1.63 | 0 | 0 | 0 | 0 | 0 | 0 |
| **-6** | 0.67 | 4.88 | 0.93 | 0 | 0 | 0 | 0 | 0 |
| **-5** | 1.34 | 4.07 | 2.78 | 1.57 | 1.2 | 0 | 0 | 0 |
| **-4** | 2.92 | 8.94 | 6.94 | 4.71 | 0 | 0 | 0 | 0 |
| **-3** | 5.51 | 14.63 | 10.19 | 9.42 | 2.41 | 1.96 | 0 | 0 |
| **-2** | 11.78 | 21.14 | 16.67 | 14.14 | 12.05 | 10.29 | 4.71 | 1.89 |
| **-1** | 16.46 | 16.26 | 16.67 | 21.47 | 18.07 | 14.71 | 15.71 | 9.43 |
| **0** | 21.64 | 23.58 | 16.2 | 13.09 | 25.9 | 26.96 | 25.65 | 21.7 |
| **1** | 18.88 | 4.88 | 23.15 | 20.42 | 18.67 | 18.63 | 17.8 | 26.42 |
| **2** | 12.61 | 0 | 6.48 | 12.57 | 14.46 | 15.2 | 19.9 | 18.87 |
| **3** | 4.34 | 0 | 0 | 2.62 | 5.42 | 7.35 | 8.38 | 6.6 |
| **4** | 2.59 | 0 | 0 | 0 | 1.81 | 3.43 | 4.71 | 11.32 |
| **5** | 0.92 | 0 | 0 | 0 | 0 | 1.47 | 2.62 | 2.83 |
| **6** | 0.17 | 0 | 0 | 0 | 0 | 0 | 0.52 | 0.94 |
| N | 1197 | 123 | 216 | 191 | 166 | 204 | 191 | 106 |
| M | -0.07 | -1.94 | -0.92 | -0.51 | 0.16 | 0.51 | 0.93 | 1.36 |
| SD | 2.01 | 1.90 | 1.89 | 1.86 | 1.65 | 1.69 | 1.68 | 1.68 |

*Each cell contains the percent of participants reporting, with columns summing to 100%.*

Table S7. Effect of change in subjective social status (SSC & SSS) on emotion perception of individuals and groups. (preregistered H4)

|  | **SSC** | | **SSS** | |
| --- | --- | --- | --- | --- |
|  | **Individual emotion perception task (GERT-S)** | **Group emotion perception task (EET)** | **Individual emotion perception task (GERT-S)** | **Group emotion perception task (EET)** |
| **Intercept** | 67.61*** | 1.03*** | 67.62*** | 1.03*** |
|  | [60.94, 74.28] | [0.74, 1.32] | [60.94, 74.3] | [0.74, 1.32] |
| **Change in social status** | -0.42 | -0.03^t^ | -0.14 | -0.01 |
|  | [-1.04, 0.21] | [-0.05, 0.001] | [-0.48, 0.2] | [-0.02, 0.01] |
| **Age** | -0.06* | 0.01*** | -0.06* | 0.01*** |
|  | [-0.11, -0.01] | [0.01, 0.01] | [-0.11, -0.01] | [0.01, 0.01] |
| **Female** | 1.8** | -0.04 | 1.81** | -0.04 |
|  | [0.49, 3.12] | [-0.1, 0.02] | [0.5, 3.13] | [-0.1, 0.02] |
| **White** | 2.03** | -0.03 | 2.06** | -0.02 |
|  | [0.52, 3.54] | [-0.09, 0.04] | [0.55, 3.58] | [-0.09, 0.04] |
| **Democrat** | 1.84** | -0.04 | 1.82** | -0.04 |
|  | [0.53, 3.15] | [-0.09, 0.02] | [0.51, 3.13] | [-0.09, 0.02] |
| **Agreeableness** | -0.3 | 0.01 | -0.3 | 0.01 |
|  | [-1.1, 0.5] | [-0.02, 0.05] | [-1.11, 0.5] | [-0.02, 0.05] |
| **Sense of Power** | -2.03** | 0.01 | -2.03** | 0.01 |
|  | [-3.5, -0.56] | [-0.05, 0.08] | [-3.5, -0.56] | [-0.05, 0.08] |
|  |  |  |  |  |

*Note: ^t^p < .10, * p < .05, ** p < .01, *** p < .001; Table contains estimate, significance, [95% CI]. The effect of subjective social status on emotion perception was tested using separate models, each containing one subjective social status measure. Column headings indicate which subjective social status measure was included in the model.*

Table S8. Effect of change in subjective social status (MSSS) on emotion perception of individuals for subset of participants in the middle of the MSSS ladder (rungs 4-6)

|  | **Individual emotion perception task (GERT-S)** | | |
| --- | --- | --- | --- |
|  | **Model 1** | **Model 2** | **Model 3** |
| **Intercept** | 67.74*** | 65.42*** | 66.5*** |
|  | [56.77, 78.7] | [56.01, 74.83] | [55.54, 77.45] |
| **Age** | -0.05 | -0.04 | -0.04 |
|  | [-0.12, 0.02] | [-0.11, 0.03] | [-0.11, 0.03] |
| **Female** | 1.16 | 1.2 | 1.18 |
|  | [-0.73, 3.06] | [-0.69, 3.08] | [-0.71, 3.06] |
| **White** | 3.4** | 2.98** | 2.99** |
|  | [1.25, 5.54] | [0.82, 5.14] | [0.83, 5.15] |
| **Democrat** | 3.34*** | 3.42*** | 3.42*** |
|  | [1.47, 5.2] | [1.56, 5.27] | [1.57, 5.28] |
| **Agreeableness** | -0.24 | -0.33 | -0.33 |
|  | [-1.43, 0.95] | [-1.52, 0.86] | [-1.52, 0.85] |
| **Sense of Power** | -1.94^t^ | -1.97^t^ | -1.97^t^ |
|  | [-4.02, 0.14] | [-4.04, 0.1] | [-4.04, 0.1] |
| **Current subjective social status (MSSS)** | -0.55 |  | -0.21 |
|  | [-1.64, 0.54] |  | [-1.33, 0.9] |
| **Change in Subjective social status (MSSS)** |  | -0.69** | -0.67* |
|  |  | [-1.21, -0.17] | [-1.2, -0.13] |

*Note: ^t^p < .10, * p < .05, ** p < .01, *** p < .001; Table contains estimate, significance, [95% CI].*

Supplemental Table S9a. Effect of subjective social status on accuracy in the group emotion perception task (exploratory analysis of H3)

|  | **MSSS** | | **SSC** | | **SSS** | |
| --- | --- | --- | --- | --- | --- | --- |
|  | **Restricted Model** | **Full Model** | **Restricted Model** | **Full Model** | **Restricted Model** | **Full Model** |
| **Intercept** | 46.33***  [38.64, 54.02] | 46.52***  [38.71, 54.32] | 45.56***  [37.76, 53.35] | 45.98***  [38.08, 53.88] | 48.18***  [40.55, 55.81] | 47.6***  [39.82, 55.39] |
| **Subjective social status measure** | 0.29  [-0.1, 0.68] | 0.18  [-0.36, 0.73] | 0.76^t^  [-0.04, 1.57] | 0.66  [-0.53, 1.85] | -0.09  [-0.51, 0.34] | -0.44  [-0.98, 0.09] |
| **Age** | -0.16***  [-0.22, -0.11] | -0.16***  [-0.22, -0.1] | -0.16***  [-0.22, -0.11] | -0.16***  [-0.22, -0.1] | -0.16***  [-0.22, -0.11] | -0.16***  [-0.22, -0.1] |
| **Female** | 1.56*  [0.09, 3.02] | 1.52*  [0.01, 3.02] | 1.58*  [0.12, 3.05] | 1.53*  [0.03, 3.03] | 1.51*  [0.04, 2.97] | 1.55*  [0.05, 3.05] |
| **White** | 0.03  [-1.66, 1.71] | 0.01  [-1.68, 1.69] | 0.002  [-1.68, 1.68] | 0.001  [-1.68, 1.69] | 0.12  [-1.57, 1.8] | 0.07  [-1.61, 1.75] |
| **Democrat** | 0.64  [-0.82, 2.09] | 0.71  [-0.76, 2.18] | 0.59  [-0.87, 2.04] | 0.67  [-0.8, 2.15] | 0.69  [-0.77, 2.14] | 0.69  [-0.78, 2.16] |
| **Agreeableness** | -0.31  [-1.19, 0.58] | -0.32  [-1.21, 0.57] | -0.27  [-1.16, 0.62] | -0.29  [-1.18, 0.6] | -0.32  [-1.22, 0.57] | -0.37  [-1.26, 0.53] |
| **Sense of Power** | -0.04  [-1.68, 1.59] | -0.03  [-1.67, 1.61] | -0.02  [-1.66, 1.61] | -0.01  [-1.65, 1.62] | -0.09  [-1.72, 1.55] | -0.03  [-1.67, 1.6] |
| **Income for self** |  | 0.15  [-0.31, 0.61] |  | 0.08  [-0.39, 0.55] |  | 0.42^t^  [-0.02, 0.42] |
| **Income for others in household** |  | 0.23  [-0.18, 0.63] |  | 0.15  [-0.28, 0.59] |  | 0.42*  [0.04, 0.81] |
| **Education for self** |  | -0.15  [-0.68, 0.38] |  | -0.17  [-0.7, 0.36] |  | -0.08  [-0.61, 0.44] |

*Note:  ^t^p < .10, * p < .05, ** p < .01, *** p < .001; Table contains estimate, significance, [95% CI].*

*.*

Supplemental Table S9b. Effect of the change in subjective social status from childhood to adulthood on accuracy in the group emotion perception task (exploratory analysis of H4)

|  | **Change in social status (MSSS)** | **Change in social status (SSC)** | **Change in social status (SSS)** |
| --- | --- | --- | --- |
| **Intercept** | 47.82***  [40.4, 55.24] | 47.76***  [40.35, 55.16] | 47.82***  [40.39, 55.25] |
| **Change in social status** | 0.25  [-0.11, 0.61] | 0.91*  [0.22, 1.6] | 0.03  [-0.34, 0.41] |
| **Age** | -0.17***  [-0.22, -0.11] | -0.17***  [-0.22, -0.11] | -0.16***  [-0.22, -0.11] |
| **Female** | 1.53*  [0.07, 3] | 1.57*  [0.11, 3.02] | 1.53*  [0.06, 2.99] |
| **White** | 0.18  [-1.5, 1.87] | 0.21  [-1.47, 1.89] | 0.1  [-1.59, 1.78] |
| **Democrat** | 0.65  [-0.81, 2.1] | 0.6  [-0.85, 2.05] | 0.68  [-0.78, 2.13] |
| **Agreeableness** | -0.3  [-1.19, 0.59] | -0.26  [-1.15, 0.62] | -0.3  [-1.2, 0.59] |
| **Sense of Power** | -0.07  [-1.7, 1.56] | -0.08  [-1.71, 1.56] | -0.08  [-1.72, 1.55] |

*Note:  ^t^p < .10, * p < .05, ** p < .01, *** p < .001; Table contains estimate, significance, [95% CI].*

Supplemental Table S10a. Effect of subjective social status on the alternative scoring of the individual emotion perception task (exploratory analysis of H3)

|  | **MSSS** | | **SSC** | | **SSS** | |
| --- | --- | --- | --- | --- | --- | --- |
|  | **Restricted Model** | **Full Model** | **Restricted Model** | **Full Model** | **Restricted Model** | **Full Model** |
| **Intercept** | 51.48***  [47.61, 55.35] | 50.57***  [46.66, 54.48] | 50.95***  [47.01, 54.88] | 50.24***  [46.28, 54.21] | 50.7***  [46.86, 54.55] | 50.05***  [46.14, 53.96] |
| **Subjective social status measure** | -0.24*  [-0.43, -0.04] | -0.29*  [-0.56, -0.02] | -0.23  [-0.64, 0.18] | -0.17  [-0.77, 0.43] | -0.11  [-0.32, 0.11] | -0.02  [-0.29, 0.25] |
| **Age** | -0.03^t^  [-0.05, 0.001] | -0.02  [-0.05, 0.01] | -0.03*  [-0.06, -0.0001] | -0.02  [-0.05, 0.01] | -0.03^t^  [-0.06, 0.0004] | -0.02  [-0.05, 0.01] |
| **Female** | 1.2**  [0.46, 1.93] | 0.91*  [0.16, 1.67] | 1.21**  [0.47, 1.94] | 0.89*  [0.13, 1.64] | 1.21**  [0.47, 1.95] | 0.89*  [0.13, 1.64] |
| **White** | 1.34**  [0.49, 2.18] | 1.3**  [0.46, 2.15] | 1.31**  [0.46, 2.16] | 1.3**  [0.45, 2.14] | 1.31**  [0.46, 2.16] | 1.29**  [0.45, 2.14] |
| **Democrat** | 1.12**  [0.39, 1.85] | 1.07**  [0.33, 1.81] | 1.11**  [0.38, 1.85] | 1.08**  [0.34, 1.82] | 1.09**  [0.36, 1.82] | 1.07**  [0.33, 1.81] |
| **Agreeableness** | -0.22  [-0.67, 0.23] | -0.28  [-0.72, 0.17] | -0.23  [-0.68, 0.22] | -0.29  [-0.74, 0.16] | -0.24  [-0.69, 0.21] | -0.29  [-0.74, 0.16] |
| **Sense of Power** | -1.33**  [-2.15, -0.5] | -1.36**  [-2.18, -0.54] | -1.31**  [-2.14, -0.49] | -1.34**  [-2.16, -0.52] | -1.3**  [-2.12, -0.47] | -1.33**  [-2.15, -0.51] |
| **Income for self** |  | -0.17  [-0.40, 0.06] |  | -0.27*  [-0.51, -0.03] |  | -0.3**  [-0.52, -0.08] |
| **Income for others in household** |  | 0.2^t^  [-0.01, 0.4] |  | 0.13  [-0.09, 0.34] |  | 0.1  [-0.1, 0.29] |
| **Education for self** |  | 0.32*  [0.05, 0.59] |  | 0.28*  [0.01, 0.54] |  | 0.27*  [0.003, 0.53] |

*Note:  ^t^p < .10, * p < .05, ** p < .01, *** p < .001; Table contains estimate, significance, [95% CI].*

Supplemental Table S10b. Effect of subjective social status on the alternative scoring of the individual emotion perception task (exploratory analysis of H4)

|  | **Change in social status (MSSS)** | **Change in social status** **(SSC)** | **Change in social status (SSS)** |
| --- | --- | --- | --- |
| **Intercept** | 50.27***  [46.53, 54.01] | 50.28***  [46.54, 54.02] | 50.29***  [46.54, 54.03] |
| **Change in social status** | -0.2*  [-0.38, -0.02] | -0.24  [-0.59, 0.11] | -0.09  [-0.28, 0.1] |
| **Age** | -0.03^t^  [-0.05, 0.002] | -0.03^t^  [-0.05, 0.001] | -0.03^t^  [-0.05, 0.001] |
| **Female** | 1.22**  [0.48, 1.95] | 1.21**  [0.48, 1.95] | 1.22**  [0.48, 1.95] |
| **White** | 1.21**  [0.36, 2.06] | 1.25**  [0.4, 2.1] | 1.27**  [0.42, 2.12] |
| **Democrat** | 1.11**  [0.38, 1.84] | 1.1**  [0.37, 1.84] | 1.09**  [0.36, 1.83] |
| **Agreeableness** | -0.22  [-0.67, 0.22] | -0.23  [-0.68, 0.22] | -0.23  [-0.68, 0.22] |
| **SensePower** | -1.3**  [-2.13, -0.48] | -1.3**  [-2.12, -0.47] | -1.29**  [-2.12, -0.47] |

*Note:  ^t^p < .10, * p < .05, ** p < .01, *** p < .001; Table contains estimate, significance, [95% CI].*

Supplemental Table S11a. Effect of subjective social status controlling for performance in the nonsocial perception task (exploratory analysis of H3)

|  | **Emotion Perception for Individuals** | | | | | | **Emotion Perception for Groups (precision)** | | | | | |
| --- | --- | --- | --- | --- | --- | --- | --- | --- | --- | --- | --- | --- |
|  | **MSSS** | | **SSC** | | **SSS** | | **MSSS** | | **SSC** | | **SSS** | |
|  | **Restricted Model** | **Full Model** | **Restricted Model** | **Full Model** | **Restricted Model** | **Full Model** | **Restricted Model** | **Full Model** | **Restricted Model** | **Full Model** | **Restricted Model** | **Full Model** |
| **Intercept** | 71.65***  [64.8, 78.51] | 70.13***  [63.19, 77.07] | 70.61***  [63.64, 77.58] | 69.48***  [62.44, 76.53] | 70.1***  [63.29, 76.92] | 69.16***  [62.22, 76.11] | 0.86***  [0.58, 1.14] | 0.85***  [0.56, 1.13] | 0.87***  [0.59, 1.16] | 0.85***  [0.56, 1.14] | 0.85***  [0.57, 1.13] | 0.84***  [0.55, 1.13] |
| **Subjective social status measure** | -0.43*  [-0.78, -0.09] | -0.54*  [-1.02, -0.06] | -0.39  [-1.11, 0.32] | -0.23  [-1.29, 0.82] | -0.16  [-0.54, 0.21] | 0.01  [-0.47, 0.48] | -0.01  [-0.02, 0.01] | -0.002  [-0.02, 0.02] | -0.01  [-0.04, 0.01] | -0.005  [-0.05, 0.04] | -0.005  [-0.02, 0.01] | 0.0004  [-0.02, 0.02] |
| **Age** | -0.05*  [-0.1, -0.002] | -0.04  [-0.09, 0.01] | -0.05*  [-0.1, -0.003] | -0.04  [-0.09, 0.01] | -0.05*  [-0.1, -0.002] | -0.04  [-0.09, 0.01] | 0.01***  [0.004, 0.01] | 0.01***  [0.004, 0.01] | 0.01***  [0.004, 0.01] | 0.01***  [0.004, 0.01] | 0.01***  [0.004, 0.01] | 0.01***  [0.004, 0.01] |
| **Female** | 1.41*  [0.11, 2.72] | 0.98  [-0.36, 2.32] | 1.43*  [0.12, 2.74] | 0.92  [-0.41, 2.26] | 1.44*  [0.13, 2.75] | 0.92  [-0.42, 2.26] | -0.0001  [-0.05, 0.05] | 0.002  [-0.05, 0.06] | -0.001  [-0.05, 0.05] | 0.002  [-0.05, 0.06] | -0.0001  [-0.05, 0.05] | 0.002  [-0.05, 0.06] |
| **White** | 2.02**  [0.53, 3.52] | 1.98**  [0.49, 3.47] | 1.97*  [0.47, 3.47] | 1.96*  [0.46, 3.45] | 1.97*  [0.47, 3.47] | 1.95*  [0.46, 3.44] | -0.005  [-0.07, 0.06] | -0.004  [-0.07, 0.06] | -0.005  [-0.07, 0.06] | -0.004  [-0.07, 0.06] | -0.01  [-0.07, 0.06] | -0.004  [-0.07, 0.06] |
| **Democrat** | 1.67*  [0.37, 2.96] | 1.61*  [0.3, 2.91] | 1.65*  [0.35, 2.95] | 1.62*  [0.32, 2.93] | 1.61*  [0.31, 2.91] | 1.61*  [0.3, 2.91] | -0.02  [-0.07, 0.04] | -0.02  [-0.07, 0.03] | -0.02  [-0.07, 0.04] | -0.02  [-0.07, 0.03] | -0.02  [-0.07, 0.04] | -0.02  [-0.07, 0.03] |
| **Agreeableness** | -0.16  [-0.95, 0.63] | -0.25  [-1.04, 0.54] | -0.17  [-0.97, 0.62] | -0.28  [-1.07, 0.52] | -0.18  [-0.98, 0.61] | -0.27  [-1.06, 0.53] | 0.002  [-0.03, 0.03] | 0.003  [-0.03, 0.04] | 0.002  [-0.03, 0.03] | 0.003  [-0.03, 0.04] | 0.002  [-0.03, 0.03] | 0.003  [-0.03, 0.04] |
| **Sense of Power** | -1.82*  [-3.27, -0.36] | -1.87*  [-3.32, -0.42] | -1.78*  [-3.24, -0.32] | -1.84*  [-3.29, -0.38] | -1.76*  [-3.22, -0.3] | -1.83*  [-3.28, -0.37] | -0.02  [-0.08, 0.04] | -0.02  [-0.08, 0.04] | -0.02  [-0.08, 0.04] | -0.02  [-0.08, 0.04] | -0.02  [-0.08, 0.04] | -0.02  [-0.08, 0.04] |
| **Income for self** |  | -0.25  [-0.66, 0.15] |  | -0.45*  [-0.87, -0.03] |  | -0.51*  [-0.90, -0.12] |  | -0.01  [-0.02, 0.01] |  | -0.01  [-0.02, 0.01] |  | -0.01  [-0.02, 0.01] |
| **Income for others in household** |  | 0.34^t^  [-0.02, 0.7] |  | 0.19  [-0.19, 0.58] |  | 0.14  [-0.2, 0.48] |  | -0.01  [-0.03, 0.004] |  | -0.01  [-0.03, 0.01] |  | -0.01  [-0.03, 0.003] |
| **Education for self** |  | 0.49*  [0.02, 0.96] |  | 0.41^t^  [-0.06, 0.88] |  | 0.39  [-0.08, 0.85] |  | 0.01  [-0.01, 0.03] |  | 0.01  [-0.01, 0.03] |  | 0.01  [-0.01, 0.03] |
| **EET Control task** | -2.93***  [-4.03, -1.84] | -2.78***  [-3.88, -1.68] | -2.96***  [-4.06, -1.86] | -2.85***  [-3.95, -1.75] | -2.94***  [-4.04, -1.84] | -2.86***  [-3.96, -1.76] | 0.31***  [0.27, 0.36] | 0.31***  [0.27, 0.36] | 0.31***  [0.27, 0.36] | 0.31***  [0.27, 0.36] | 0.31***  [0.27, 0.36] | 0.31***  [0.27, 0.36] |

*Note:  ^t^p < .10, * p < .05, ** p < .01, *** p < .001; Table contains estimate, significance, [95% CI].*

Supplemental Table S11b. Effect of change in subjective social status controlling for performance in the nonsocial perception task (exploratory analysis of H4)

|  | Emotion Perception for Individuals | | | Emotion Perception for Groups (precision) | | |
| --- | --- | --- | --- | --- | --- | --- |
|  | **Change in social status (MSSS)** | **Change in social status (SSC)** | **Change in social status (SSS)** | **Change in social status (MSSS)** | **Change in social status (SSC)** | **Change in social status (SSS)** |
| **Intercept** | 69.43***  [62.81, 76.06] | 69.47***  [62.84, 76.11] | 69.48***  [62.84, 76.11] | 0.83***  [0.56, 1.1] | 0.83***  [0.56, 1.1] | 0.83***  [0.56, 1.1] |
| **Change in social status** | -0.34*  [-0.65, -0.02] | -0.41  [-1.03, 0.2] | -0.13  [-0.46, 0.21] | -0.01  [-0.02, 0.01] | -0.03*  [-0.05, -0.001] | -0.01  [-0.02, 0.01] |
| **Age** | -0.05^t^  [-0.1, 0.001] | -0.05*  [-0.1, -0.001] | -0.05*  [-0.1, -0.001] | 0.01***  [0.004, 0.01] | 0.01***  [0.004, 0.01] | 0.01***  [0.004, 0.01] |
| **Female** | 1.45*  [0.14, 2.75] | 1.44*  [0.13, 2.75] | 1.45*  [0.14, 2.76] | 0.0003  [-0.05, 0.05] | -0.001  [-0.05, 0.05] | -0.0002  [-0.05, 0.05] |
| **White** | 1.81*  [0.31, 3.3] | 1.87*  [0.37, 3.37] | 1.91*  [0.41, 3.4] | -0.01  [-0.07, 0.05] | -0.01  [-0.07, 0.05] | -0.01  [-0.07, 0.05] |
| **Democrat** | 1.65*  [0.35, 2.94] | 1.64*  [0.34, 2.93] | 1.61*  [0.32, 2.91] | -0.02  [-0.07, 0.04] | -0.01  [-0.07, 0.04] | -0.02  [-0.07, 0.04] |
| **Agreeableness** | -0.16  [-0.95, 0.63] | -0.18  [-0.97, 0.62] | -0.17  [-0.97, 0.62] | 0.002  [-0.03, 0.03] | 0.001  [-0.03, 0.03] | 0.001  [-0.03, 0.03] |
| **Sense of Power** | -1.77*  [-3.23, -0.32] | -1.76*  [-3.21, -0.3] | -1.75*  [-3.21, -0.29] | -0.02  [-0.08, 0.04] | -0.02  [-0.08, 0.04] | -0.02  [-0.08, 0.04] |
| **EET Control task** | -2.93***  [-4.03, -1.84] | -2.96***  [-4.06, -1.86] | -2.95***  [-4.05, -1.86] | 0.31***  [0.27, 0.36] | 0.31***  [0.27, 0.36] | 0.31***  [0.27, 0.36] |

*Note:  ^t^p < .10, * p < .05, ** p < .01, *** p < .001; Table contains estimate, significance, [95% CI].*

Table S12. Distribution of responses used to create alternative GERT score.

| correct emotion | Pride | Joy | Amusement | Pleasure | Relief | Interest | Surprise | Anxiety | Fear | Despair | Sadness | Disgust | Irritation | Anger |
| --- | --- | --- | --- | --- | --- | --- | --- | --- | --- | --- | --- | --- | --- | --- |
| Pride 1 | 17.63 | 32.66 | 29.74 | 7.18 | 1.25 | 8.02 | 3.34 | 0.08 | 0 | 0 | 0 | 0 | 0.08 | 0 |
| Pride 2 | 35.84 | 3.01 | 10.44 | 3.68 | 2.84 | 27.07 | 2.42 | 3.09 | 0.17 | 0.92 | 0.92 | 1.5 | 6.18 | 1.92 |
| Pride 3 | 46.62 | 17.38 | 17.46 | 9.69 | 1.34 | 7.02 | 0.25 | 0 | 0 | 0.08 | 0 | 0.08 | 0.08 | 0 |
| Joy 1 | 2.59 | 58.9 | 14.62 | 2.34 | 3.68 | 0.75 | 13.87 | 1.17 | 0.92 | 0.5 | 0.17 | 0.08 | 0.33 | 0.08 |
| Joy 2 | 4.34 | 65.08 | 4.68 | 4.09 | 2.42 | 1.75 | 17.46 | 0.08 | 0 | 0.08 | 0 | 0 | 0 | 0 |
| Joy 3 | 0.92 | 74.02 | 14.54 | 2.17 | 0.75 | 0.5 | 6.6 | 0.25 | 0 | 0 | 0.17 | 0 | 0 | 0.08 |
| Amusement 1 | 0.58 | 11.78 | 71.76 | 1.92 | 2.84 | 0.5 | 5.51 | 0.75 | 0.84 | 2.17 | 1 | 0.08 | 0.17 | 0.08 |
| Amusement 2 | 1.25 | 24.73 | 68.42 | 2.34 | 1.84 | 0.08 | 0.17 | 0.42 | 0.17 | 0.17 | 0.42 | 0 | 0 | 0 |
| Amusement 3 | 0.33 | 15.12 | 81.04 | 1.92 | 1 | 0.17 | 0.33 | 0 | 0 | 0 | 0 | 0 | 0.08 | 0 |
| Pleasure 1 | 2.59 | 10.19 | 8.6 | 68.25 | 7.77 | 1.92 | 0.5 | 0.08 | 0 | 0 | 0.08 | 0 | 0 | 0 |
| Pleasure 2 | 0.92 | 0.84 | 0.67 | 91.98 | 4.43 | 0.92 | 0 | 0 | 0 | 0 | 0.08 | 0 | 0.17 | 0 |
| Pleasure 3 | 2.76 | 3.59 | 4.68 | 75.86 | 11.78 | 0.92 | 0.08 | 0 | 0 | 0 | 0 | 0.25 | 0.08 | 0 |
| Relief 1 | 1.84 | 1.25 | 2.76 | 13.37 | 72.26 | 7.02 | 0.67 | 0.33 | 0 | 0.25 | 0 | 0.08 | 0.17 | 0 |
| Relief 2 | 0.33 | 0.17 | 0.33 | 26.82 | 62.07 | 1 | 0.25 | 1.34 | 0.58 | 2.26 | 1.5 | 0.33 | 2.42 | 0.58 |
| Relief 3 | 2.42 | 0.58 | 0.5 | 8.77 | 76.19 | 5.76 | 0.17 | 1.25 | 0 | 1.5 | 0.92 | 0.17 | 1.67 | 0.08 |
| Interest 1 | 2.09 | 0 | 1.75 | 0.84 | 3.17 | 63.91 | 3.26 | 3.84 | 0.75 | 0.5 | 0.25 | 1.5 | 17.54 | 0.58 |
| Interest 2 | 0.17 | 0 | 0.42 | 0.67 | 1.17 | 88.89 | 2.67 | 3.43 | 0.33 | 0.42 | 0.25 | 0.67 | 0.84 | 0.08 |
| Interest 3 | 0.33 | 0 | 0.75 | 0.42 | 0.58 | 78.86 | 3.93 | 3.51 | 0.33 | 1 | 0.92 | 2.84 | 6.43 | 0.08 |
| Surprise 1 | 0.08 | 0 | 0.33 | 0.17 | 1.25 | 20.05 | 16.12 | 34.75 | 14.95 | 4.59 | 3.84 | 0.75 | 2.84 | 0.25 |
| Surprise 2 | 0.17 | 0.17 | 16.46 | 0.5 | 2.92 | 2.59 | 32.08 | 3.59 | 0.42 | 1.5 | 0.42 | 15.37 | 23.81 | 0 |
| Surprise 3 | 0.25 | 0.08 | 0.17 | 0.25 | 1.25 | 15.46 | 74.35 | 3.68 | 0.67 | 1.09 | 0.08 | 0.67 | 1.92 | 0.08 |
| Anxiety 1 | 0.08 | 0 | 0.08 | 0 | 1 | 31.33 | 13.87 | 36.42 | 14.95 | 0.58 | 0.33 | 0.25 | 1.09 | 0 |
| Anxiety 2 | 0.17 | 0.17 | 0.25 | 0.25 | 6.43 | 3.68 | 2.26 | 47.62 | 14.54 | 7.85 | 13.53 | 0.84 | 2.42 | 0 |
| Anxiety 3 | 0 | 0.08 | 0.08 | 0 | 0.33 | 3.09 | 5.85 | 31.66 | 43.86 | 4.09 | 1.59 | 4.93 | 3.68 | 0.75 |
| Fear 1 | 0.17 | 0.08 | 0.08 | 0 | 0.25 | 0.33 | 5.6 | 13.62 | 56.22 | 8.19 | 0.17 | 0.75 | 3.26 | 11.28 |
| Fear 2 | 0 | 0 | 0.08 | 0.08 | 0 | 0.08 | 3.34 | 5.76 | 52.3 | 4.76 | 0.17 | 1.17 | 4.34 | 27.9 |
| Fear 3 | 0.08 | 0.17 | 0.25 | 0 | 0.33 | 0.58 | 19.3 | 19.13 | 33.25 | 4.09 | 0.25 | 1.75 | 12.11 | 8.69 |
| Despair 1 | 0.17 | 0 | 0.08 | 0.33 | 0.17 | 0.08 | 0.17 | 7.1 | 14.95 | 50.13 | 24.06 | 0.33 | 1.84 | 0.58 |
| Despair 2 | 0 | 0.17 | 0.17 | 0 | 0.25 | 0.08 | 0.08 | 1.09 | 3.43 | 75.19 | 13.78 | 1.67 | 1 | 3.09 |
| Despair 3 | 0.08 | 0 | 0.17 | 0 | 0 | 0.08 | 0.25 | 5.01 | 14.2 | 48.37 | 19.05 | 2.84 | 2.17 | 7.77 |
| Sadness 1 | 0.17 | 0 | 0.08 | 0 | 0.58 | 0.75 | 0.08 | 4.18 | 3.51 | 18.05 | 72.1 | 0.42 | 0.08 | 0 |
| Sadness 2 | 1.17 | 0 | 0.33 | 0.67 | 3.59 | 4.09 | 0.5 | 3.76 | 0.75 | 15.29 | 50.63 | 6.1 | 12.78 | 0.33 |
| Sadness 3 | 0.25 | 0 | 0.08 | 0.08 | 0.42 | 0.75 | 0.17 | 2.17 | 0.58 | 22.89 | 69.84 | 0.92 | 1.84 | 0 |
| Disgust 1 | 0.17 | 0 | 0 | 0.17 | 0.33 | 0.42 | 0.25 | 3.76 | 0.67 | 12.36 | 23.22 | 54.39 | 4.26 | 0 |
| Disgust 2 | 0.17 | 0 | 0 | 0.08 | 0.42 | 0.67 | 0.33 | 17.96 | 6.43 | 21.97 | 26.65 | 18.88 | 6.27 | 0.17 |
| Disgust 3 | 0 | 0.08 | 0 | 0.08 | 0.17 | 0.08 | 0.5 | 1.25 | 4.68 | 5.18 | 1.75 | 85.46 | 0.67 | 0.08 |
| Irritation 1 | 3.43 | 1 | 2.26 | 0.33 | 4.43 | 26.07 | 1.25 | 17.38 | 2.34 | 3.26 | 0.5 | 1.25 | 36.26 | 0.25 |
| Irritation 2 | 0.25 | 0 | 0.17 | 0.33 | 2.51 | 2.09 | 0.08 | 6.18 | 0.33 | 5.01 | 5.93 | 7.77 | 68.67 | 0.67 |
| Irritation 3 | 1.84 | 0 | 0.42 | 0.08 | 2.26 | 9.94 | 0.25 | 0.84 | 0.08 | 1.34 | 1 | 9.77 | 69.42 | 2.76 |
| Anger 1 | 0.08 | 0 | 0.08 | 0.08 | 0 | 0.17 | 0.08 | 0.25 | 0.08 | 0.17 | 0.08 | 1.59 | 13.53 | 83.79 |
| Anger 2 | 0.08 | 0 | 0 | 0 | 0.17 | 0.08 | 0 | 0.33 | 0.25 | 0.75 | 0.17 | 4.26 | 19.3 | 74.6 |
| Anger 3 | 0 | 0.08 | 0.08 | 0.08 | 0.17 | 0.17 | 2.17 | 1.67 | 0.75 | 0.92 | 0.08 | 2.26 | 18.88 | 72.68 |

Note: Each row represents a video, labeled with the emotion expressed by the actor. Each column represents the response option selected. Each cell contains the percent of participants choosing the emotion indicated in the column headings. Rows sum to 100%
